# Novel Molecular Determinants of Response or Resistance to Immune Checkpoint Inhibitor Therapies in Melanoma

**DOI:** 10.3389/fimmu.2021.798474

**Published:** 2022-01-11

**Authors:** Wenjing Zhang, Yujia Kong, Yuting Li, Fuyan Shi, Juncheng Lyu, Chao Sheng, Suzhen Wang, Qinghua Wang

**Affiliations:** ^1^ Department of Health Statistics, Key Laboratory of Medicine and Health in Shandong Province, School of Public Health, Weifang Medical University, Weifang, China; ^2^ Tianjin Cancer Institute, National Clinical Research Center for Cancer, Key Laboratory of Cancer Prevention and Therapy of Tianjin, Tianjin Medical University Cancer Institute and Hospital, Tianjin, China; ^3^ Department of Epidemiology and Biostatistics, National Clinical Research Center for Cancer, Key Laboratory of Molecular Cancer Epidemiology of Tianjin, Tianjin Medical University Cancer Institute and Hospital, Tianjin, China

**Keywords:** melanoma, immunotherapy, SMGs, mutational signatures, molecular subtypes, predictive biomarkers

## Abstract

**Background:**

Immune checkpoint inhibitor (ICI) therapy dramatically prolongs melanoma survival. Currently, the identified ICI markers are sometimes ineffective. The objective of this study was to identify novel determinants of ICI efficacy.

**Methods:**

We comprehensively curated pretreatment somatic mutational profiles and clinical information from 631 melanoma patients who received blockade therapy of immune checkpoints (i.e., CTLA-4, PD-1/PD-L1, or a combination). Significantly mutated genes (SMGs), mutational signatures, and potential molecular subtypes were determined. Their association with ICI responses was assessed simultaneously.

**Results:**

We identified 27 SMGs, including four novel SMGs (*COL3A1*, *NRAS*, *NARS2*, and *DCC*) that are associated with ICI efficacy and well-known driver genes. *COL3A1* mutations were associated with improved ICI overall survival (hazard ratio (HR): 0.64, 95% CI: 0.45–0.91, *p* = 0.012), whereas immune resistance was observed in patients with *NRAS* mutations (HR: 1.42, 95% CI: 1.10–1.82, *p* = 0.006). The presence of the tobacco smoking-related signature was significantly correlated with inferior prognoses (HR: 1.42, 95% CI: 1.11–1.82, *p* = 0.005). In addition, the signature resembling that of alkylating agents and a newly discovered signature both exhibited extended prognoses (both HR < 1, *p* < 0.05). Based on the activities of the extracted 6 mutational signatures, we identified one immune subtype that was significantly associated with better ICI outcomes (HR: 0.44, 95% CI: 0.23–0.87, *p* = 0.017).

**Conclusion:**

We uncovered several novel SMGs and re-annotated mutational signatures that are linked to immunotherapy response or resistance. In addition, an immune subtype was found to exhibit favorable prognoses. Further studies are required to validate these findings.

## Introduction

The blockade of cytotoxic T lymphocyte antigen-4 (CTLA-4), programmed cell death-1 (PD-1), or its ligand PD-L1 with monoclonal antibodies (e.g., ipilimumab, pembrolizumab, or nivolumab) considerably prolongs the survival of patients with advanced or metastatic melanoma (1). The insight that inhibition of immune checkpoints can result in the reversion of inactivated T cells has dramatically changed cancer therapy patterns (2). Despite impressive durable clinical benefits, immune checkpoint inhibitors (ICIs) offer a long-term response only to a subset of patients with melanoma (3). Therefore, selecting among patients the subpopulation that will respond to ICI therapy remains a problem that needs to be urgently solved.

Initial clinical trials of anti-PD-1 showed that tumors expressing high PD-L1 levels were associated with benefits to treatment (4–6). However, further studies have reported that a greater proportion of responders were patients with negative PD-L1 expression (7–9). Neoantigens are computationally obtained based on somatic mutational profiles, and an elevated neoantigen burden (NB) has been shown to underlie the responses to ICI treatment. Tumor mutational burden (TMB) is consistently correlated with elevated benefits to ICI agents, initially in trials of melanoma and non-small cell lung cancer (NSCLC) (10–12). The association of high TMB with improved ICI response has also been observed in several other cancers (13–15). Nevertheless, TMB is an unstable indicator because it does not exhibit an association with the response in other cancers, such as renal cell cancer (16), Hodgkin’s lymphoma (17), and virally mediated Merkel-cell carcinoma (18). The above observations drive us to explore novel determinants of the benefits of checkpoint inhibition treatment.

Several recent studies have reported that mutations in single genes, such as *POLE* (19), *POLD1* (19), *PBRM1* (20), *TTN* (21), and *MUC16* (22), were correlated with favorable ICI response or survival. Nevertheless, mutations in *B2M*, which stabilize intracellular peptides on the cell surface and play a vital role in antigen presentation, were demonstrated to be associated with acquired resistance to CTLA-4 and PD-1 inhibitors in melanoma (23). Similarly, *JAK1* or *JAK2* mutations have also been linked with primary or acquired resistance to anti-PD-1 therapy in advanced melanoma and colon carcinoma (24, 25).

Specific mutational signatures, which are characteristic patterns of mutation types produced by distinct mutation processes, have been shown to be associated with ICI response (2). Lung cancer patients harboring tobacco smoking-related mutational signatures exhibited a better clinical benefit than those without such signatures (12). Tumors with a durable anti-PD-1 response displayed an accumulation of a mutational signature correlated with apolipoprotein B mRNA editing enzyme, catalytic polypeptide-like (APOBEC) (26, 27). Moreover, melanoma patients who harbored ultraviolet light exposure-related mutational signatures were more likely to experience favorable responses when receiving immune checkpoint-based therapies (27). It will be of interest to explore whether other DNA-damaging mutational signatures are linked with immunotherapy responses and to uncover novel signatures that were not previously annotated in melanoma.

Immune molecular subtypes based on multi-omics data have recently been identified in melanoma (28–30). However, most of these identified subtypes are employed to predict tumor intrinsic prognoses and cannot be used to evaluate the therapeutic effect. Current immunotherapy studies of malignant melanoma are mostly focused on somatic mutation levels, and fewer studies included continuous data (e.g., gene expression profiles). Feasibly, potential molecular subtypes could be obtained by clustering the mutational signature activities extracted from mutational profiles (31), and a further selection of immune subtypes could be achieved by evaluating the association between distinct subgroups and immunotherapy efficacy.

We hypothesized that an expanded clinically annotated melanoma cohort could more effectively be used to detect significant correlations between pretreatment genomic features and ICI efficacy. Therefore, we curated pretreatment somatic data from melanoma samples treated with ICI agents. By integrating mutational profiles and clinicopathologic characteristics across 631 samples, we aimed to identify novel significantly mutated genes (SMGs) and potential immune subtypes that are associated with response or resistance to ICI treatment and to re-annotate the mutational signatures in the setting of immunotherapy.

## Methods

### Genomic Data and Clinical Information

A total of 333,968 pretreatment whole-exome sequencing non-synonymous somatic alterations in 631 melanoma patients treated with ICIs (i.e., anti-CTLA-4, anti-PD-1/PD-L1, or combined therapy) from eight previously published studies were collected (11, 25, 27, 32–36). Mutation types in this study included missense mutations, nonsense mutations, frameshift del/ins, in frame del/ins, and splice site mutations. All somatic mutations were uniformly re-annotated using the Oncotator (37). Gene expression profiles were curated in three of eight studies (33, 34, 36). Clinicopathologic characteristics including age, sex, ICI response status, follow-up information on overall survival (OS) and progression-free survival (PFS), and ICI types of the above eight studies are shown in **Table S1**. Of the aggregated 631 melanoma patients, 627 had data regarding OS times and status, and 390 had information on PFS times and status. Other available data for all patients are shown in **Table S2**. Objective response rates (ORRs) indicate the proportion of patients with complete response (CR) or partial response (PR) status. Disease control rates (DCRs) reflect the proportion of patients who achieve a non-progressive disease status (i.e., CR, PR, and stable disease [SD]).

A total of 313 ICI-treated melanoma samples, which are subjected to the Integrated Mutation Profiling of Actionable Cancer Targets (MSK-IMPACT) assay of a targeted 468-gene panel at Memorial Sloan Kettering Cancer Center (MSKCC), were also collected for specific validation (38). Detailed clinical characteristics are illustrated in **Table S3**. Clinical information and somatic mutational profiles of 457 melanoma samples from The Cancer Genome Atlas (TCGA) were downloaded from the Genome Data Commons (https://gdc.cancer.gov).

### Identification of Significantly Mutated Genes

SMGs were identified using the MutSigCV algorithm against the hg19 genome (39). MutSigCV detects significantly enriched non-silent somatic alterations in one gene by considering the background mutation rate estimated through silent mutations. In addition to being statistically significant by this algorithm (*q* < 0.1), a putative SMG must meet the criterion of expressing in TCGA melanoma dataset (40). The mutational patterns of SMGs were visualized using the R package GenVisR (41).

### Deciphering Mutational Signatures Operative in the Genome

The algorithm published by Kim et al. (42) was applied to detect mutational signatures in the integrated melanoma cohort. The core of this method is Bayesian variant non-negative matrix factorization (NMF), which can automatically calculate the optimal number of mutational signatures and eliminate manual inspection. Specifically, NMF was applied to decompose mutation portrait matrix *A*, which contained 96 base substitution classes with trinucleotide sequence patterns. Matrix A was factorized into two non-negative matrices *W* and *H* (i.e., *A* ≈ *WH*), where *W* indicates the extracted mutational signatures and *H* represents the mutation activities of each corresponding signature. The column of matrix *A* is the count of detected signatures, and rows represent the 96 base substitution types, which are the permutation and combination of six main mutational categories (i.e., C > A, C > G, C > T, T > A, T > C, and T > G) and their surrounding adjacent bases. The rows and columns of matrix *H* indicate the individual signatures and their corresponding mutational activities, respectively. All extracted mutational signatures were then compared with the 30 annotated signatures stored in the Catalogue of Somatic Mutations in Cancer (COSMIC; version 2) based on cosine similarity. The detected mutational signatures were defined as binary variables (i.e., yes and no) in survival analyses and multivariate Cox regression models according to the principle proposed by a recent study: a signature was supposed to exist in a sample if it contributed to greater than 100 substitutions or 25% of the total mutations (43).

### Detection of Potential Molecular Subtypes

We employed consensus clustering to determine the potential molecular subtypes of the integrated melanoma patients. After obtaining the activities of extracted mutational signatures of all patients, we then used the partition around medoids (PAM) algorithm with the Euclidean distance metric and performed 500 bootstraps, each comprising 80% of patients in the aggregated cohort. The clustering number was explored from 2 to 10, and the optimal number was determined by evaluating the cluster consensus coefficient and consensus matrix. Consensus clustering analysis was conducted using the R package ConsensusClusterPlus (44).

### Estimation of Tumor Infiltration Lymphocytes

The CIBERSORT algorithm was used to calculate the proportion of infiltrating immune cell subsets in tumors, which is an analytical tool that imputes gene expression profiles and provides an estimation of the abundance of 22 human hematopoietic cell phenotypes with 547 genes from the leukocyte gene signature matrix, termed LM22 (45). The 22 cell subsets include 7 T-cell types, naive and memory B cells, plasma cells, NK cells, and myeloid subsets, which exert distinct functionalities in antitumor immune responses.

### Differential Analysis and Gene Set Enrichment Analysis

Differential expression of each gene in distinct subgroups was calculated using the R package limma (46) and edgeR (47). Especially, read counts of gene expression profiles were normalized using the calcNormFactors function in the package edgeR, and then as input to lmFit and eBayes functions in the limma package. The differential expression *t* statistics obtained from eBayes function were subsequently used to conduct gene set enrichment analysis (GSEA) implemented by R fgsea package (http://bioconductor.org/packages/release/bioc/html/fgsea.html). Cell signaling pathways and biological processes in the Kyoto Encyclopedia of Genes and Genomes (KEGG) and Gene Ontology (GO) were utilized as background datasets. The false discovery rate (FDR) and normalized enrichment score (NES) were calculated based on 1 million permutations.

### Association of Gene Mutations With Tumor Mutational Burden and Neoantigen Burden

Genome instability is markedly influenced by mutations in the genomic maintenance genes (48). Therefore, in addition to univariate analysis of the association of specific gene mutations with TMB and NB, multivariate logistic regression models with mutations in DNA damage repair genes (i.e., *BRCA1/2*, *TP53*, and *POLE*) and mismatch repair (MMR) genes (i.e., *MLH1*, *MSH2*, *MSH6*, and *PMS2*) taken into account were also conducted to control false positives. In this study, TMB was defined as the log2 transformation of total non-synonymous mutations per megabase. The neoantigen data of 340 melanoma patients were downloaded from The Cancer Immunome Atlas (TCIA; https://www.tcia.at/home).

### Statistical Analyses

Statistical analyses were performed employing R software (version 4.0.1). A genomic overview of the aggregated melanoma cohort was achieved using the maftools package (49). The Kaplan–Meier survival analyses and multivariate Cox regression models implemented by survival and forest model packages, respectively, were used to evaluate the associations of SMG mutations, the presence of mutational signatures, and potential subtypes with survival outcomes. Furthermore, the log-rank test was applied to compare the significant differences between the survival curves. The correlation of continuous and categorical variables with specific binary factors was evaluated using Wilcoxon’s rank-sum test and Fisher’s exact test, respectively. A two-sided *p*-value of less than 0.05 was considered to be statistically significant.

## Results

### Pretreatment Genomic Features and Significantly Mutated Genes Linked With Immune Checkpoint Inhibitor Response

The integrated somatic mutational profiles and clinically annotated information of 631 melanoma patients derived from eight previously published ICI studies were obtained (**Table S1**). A genomic mutation overview of the aggregated cohort is shown in **Figure S1**. Among 631 ICI-treated tumors, 193 (30.6%) showed CR/PR, 89 (14.1%) SD, 341 (54.1%) PD, and four (0.6%) mixed response, and four (0.6%) were not evaluated. Overall, 324 (51.4%) patients were treated with anti-CTLA-4, 163 (25.8%) were treated with anti-PD-1/PD-L1, and 144 (22.8%) received combined therapy (i.e., anti-CTLA-4 plus anti-PD-1/PD-L1). The median ICI OS and PFS were 19.2 and 3.5 months, respectively.

We calculated the TMB of this integrated cohort and compared it with that of 33 cancer types in TCGA. Consistent with previous observations (2), melanoma and NSCLC were two cancers with the highest TMB (**Figure S2**). We treated TMB as a continuous variable to evaluate its association with ICI efficacy. The results demonstrated that elevated TMB was significantly correlated with improved ICI OS and PFS in multivariate Cox regression models (*p* < 0.001 and *p* = 0.065, respectively; **Figures S3A, B**). In addition, we observed that high TMB was more enriched in patients with better ICI efficacy (i.e., objective response and disease control) in univariate analysis (Wilcoxon’s rank-sum test, *p* < 0.001 and *p* = 0.003, respectively; **Figures S3C, D**) and multivariate logistic models (both *p* < 0.001; **Figures S3E, F**).

**Figure 1:**
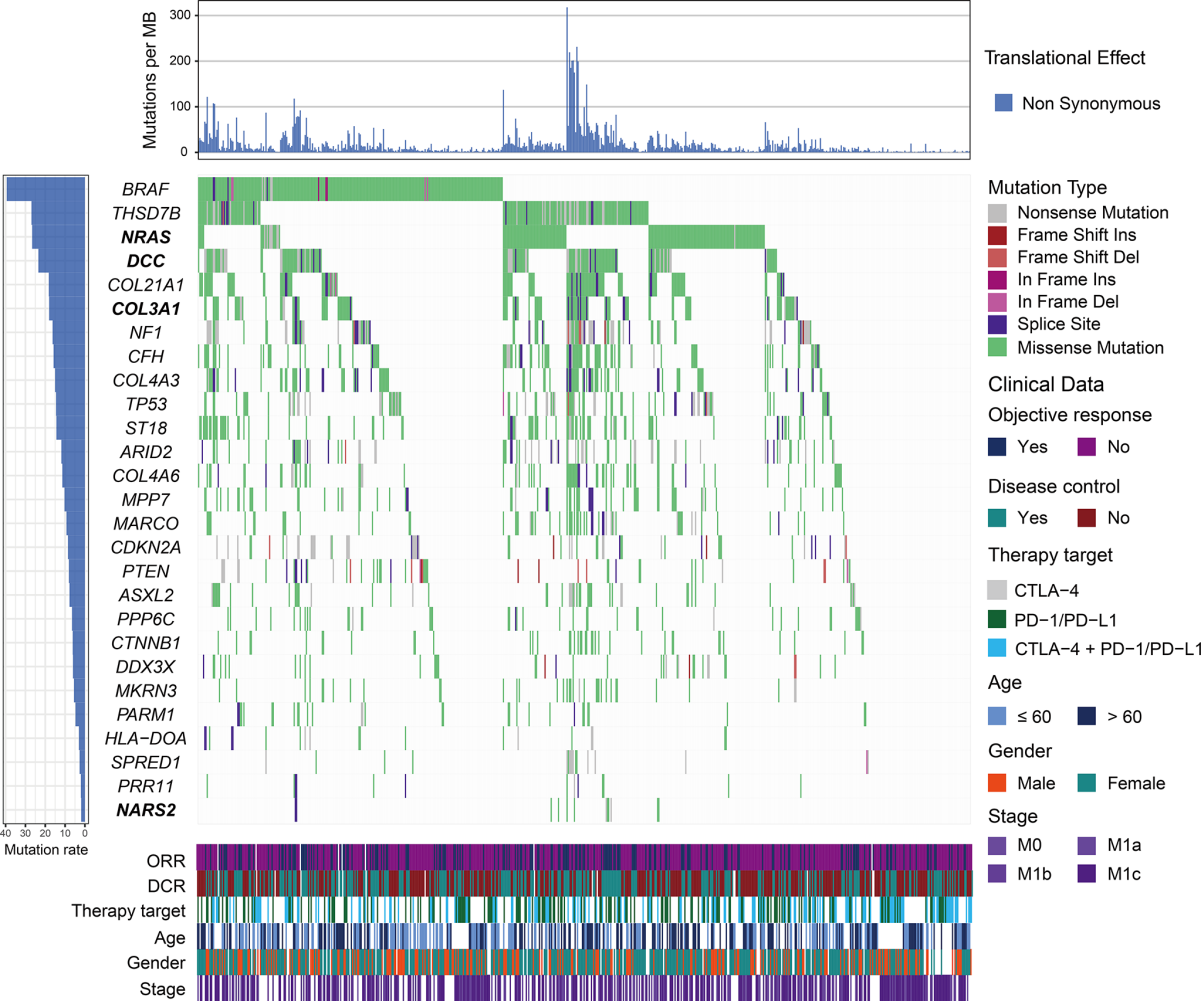
Mutational patterns of significantly mutated genes (SMGs) in melanoma. The left panel depicts the mutation rate of each SMG, the top panel represents non-synonymous mutation burden across integrated patients, the middle panel indicates mutational patterns of identified SMGs with distinct mutation types colored distinctly, and the bottom panel shows clinical characteristics such as age, gender, stage, therapy target, objective response status, and disease control status. SMGs associated with immune checkpoint inhibitor (ICI) efficacy are highlighted in bold.

We employed the MutSigCV algorithm to detect SMGs. In total, 27 SMGs were identified, including well-known driver genes (e.g., *BRAF*, *NF1*, *TP53*, *ARID2*, *PTEN*, *PPP6C*, and *DDX3X*) and several novel genes (**Figure 1** and **Table S4**). We then explored the associations of all identified SMGs with ICI OS, PFS, ORR, and DCR. We observed that numerous gene mutations exhibited a significant association with ICI efficacy (e.g., *CFH*, *MKRN3*, *NF1*, and *THSD7B*); nevertheless, the associations were not found to be significant by multivariate-adjusted analysis (**Table S4**). Finally, we identified four novel SMGs (*COL3A1*, *NRAS*, *NARS2*, and *DCC*), whose alterations were linked with ICI response or resistance (**Table S4**). The detailed mutational patterns of these four genes are shown in **Figure S4**. *COL3A1* is a member of the fibrillar collagen family that functions in extensible connective tissues such as the skin, and alterations in *COL3A1* have been demonstrated to be associated with melanoma metastasis. *NRAS* is an oncogene typically found in melanoma, and multiple targeted therapy agents have been developed for the treatment of *NRAS*-mutated melanoma. *NARS2*, mutated in 1.9% of the total patients, was found to be involved in the prognosis of neurodegenerative disorders (e.g., Alzheimer’s disease). The transmembrane protein DCC is a member of the immunoglobulin superfamily of cell adhesion molecules and functions as a tumor suppressor in several cancers, including melanoma.

### *COL3A1* Mutations Predictive of Improved Immune Checkpoint Inhibitor Survival

**Figure 2:**
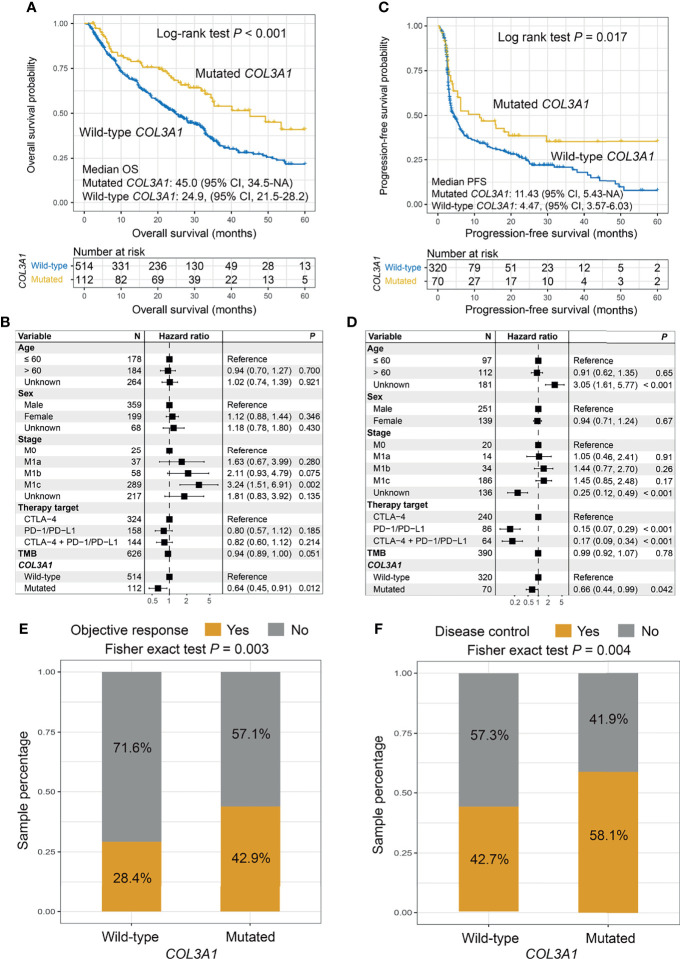
Association of *COL3A1* mutations with immune checkpoint inhibitor (ICI) survival outcome and response. Kaplan–Meier survival analyses and multivariate Cox regression models with confounding factors taken into account were conducted to evaluate the links of *COL3A1* mutations with **(A, B)** overall survival (OS) and **(C, D)** progression-free survival (PFS). *COL3A1* mutations are associated with **(E)** objective response rate (ORR) and **(F)** disease control rate (DCR).

The Kaplan–Meier analysis indicated that patients with *COL3A1* mutations showed a significantly improved ICI OS compared with patients without such mutations (median OS: 45.0 [95% CI, 34.5–NA] vs. 24.9 [95% CI, 21.5–28.2] months; log-rank test *p* < 0.001; **Figure 2A**). This association remained significant in the multivariate Cox regression model when age, sex, stage, therapy type, and TMB were taken into consideration (hazard ratio (HR): 0.64, 95% CI: 0.45–0.91, *p* = 0.012; **Figure 2B**). Consistently, an improved PFS was also observed in patients with *COL3A1* mutations in survival analysis (median PFS: 11.43 [95% CI, 5.43–NA] vs. 4.47 [95% CI, 3.57–6.03] months; log-rank test *p* = 0.017; **Figure 2C**) and multivariate analysis (HR: 0.66, 95% CI: 0.44–0.99, *p* = 0.042; **Figure 2D**). We further explored the association of *COL3A1* mutations with ICI ORR and DCR. The results suggested that *COL3A1*-mutated tumors exhibited an elevated ORR (42.9% vs. 28.4%; Fisher’s exact test *p* = 0.003; **Figure 2E**) and DCR (58.1% vs. 42.7%; Fisher’s exact test *p* = 0.004; **Figure 2F**), and marginal statistical significance was observed in the multivariate logistic regression model (*p* = 0.091 and 0.076, respectively; **Figures S5A, B**). The association of *COL3A1* mutations with ICI survival in distinct ICI types was assessed. We found that *COL3A1* mutations were associated with improved OS in anti-CTLA-4 and combined therapies (log-rank test *p* = 0.045 and 0.007, respectively; **Figures S6A, C**). In the anti-PD-1/PD-L1 therapy, a trend of better prognosis was observed in *COL3A1*-mutated patients, although it did not reach statistical significance (log-rank test *p* = 0.094; **Figure S6B**). *COL3A1* mutation associations with ORR (**Figures S6D–F**) and DCR (**Figures S6G–I**) in the three ICI types were also evaluated and illustrated. Six of seven individual cohorts showed trends of improved OS of patients with *COL3A1* mutations (**Figure S7**); the Zaretsky et al. cohort was not evaluated because it harbors only four melanoma patients. We evaluated the prognostic power of *COL3A1* mutations in TCGA melanoma cohort. No significant associations were observed between *COL3A1* mutations and OS (log-rank test *p* = 0.946, multivariate Cox *p* = 0.602; **Figures S8A, B**) and PFS (log-rank test *p* = 0.813, multivariate Cox *p* = 0.618; **Figures S8C, D**).

We further investigated the possible mechanisms underlying the *COL3A1* mutations. First, an enhanced TMB was observed in the *COL3A1*-mutated patients (Wilcoxon’s rank-sum test *p* < 0.001; **Figure S9A**). This link remained significant even after adjusting for mutations in *BRCA1/2*, *TP53*, *POLE*, and MMR genes (OR: 16.11, 95% CI: 8.23–35.46, *p* < 0.001; **Figure S9B**). Consistent results were also observed for NB in univariate analysis (Wilcoxon’s rank-sum test *p* < 0.001; **Figure S9C**) and multivariate logistic regression (OR: 4.94, 95% CI: 2.32–11.34, *p* < 0.001; **Figure S9D**). Second, immune cell infiltration analysis revealed that CD8 T cells, activated CD4 memory T cells, and resting NK cells infiltrated tumors of patients with *COL3A1* mutations (Wilcoxon’s rank-sum test all *p* < 0.05; **Figure S9E**). Noticeably, *COL3A1* mutant tumors exhibited increased infiltration of pro-inflammatory M1 macrophages (Wilcoxon’s rank-sum test *p* < 0.001; **Figure S9E**) and decreased infiltration of immune-suppressive M2 macrophages (Wilcoxon’s rank-sum test *p* = 0.011; **Figure S9E**). Third, GSEA results suggested that antigen processing and presentation-related pathways in KEGG and GO databases were enriched in patients with *COL3A1* mutations (all FDR < 0.05; **Figures S9F–J**). Collectively, favorable genomic traits and the immune microenvironment may underlie the better ICI response of *COL3A1* mutations.

### *NRAS*, *NARS2*, and *DCC* Mutations Associated With Immune Checkpoint Inhibitor Efficacy

**Figure 3:**
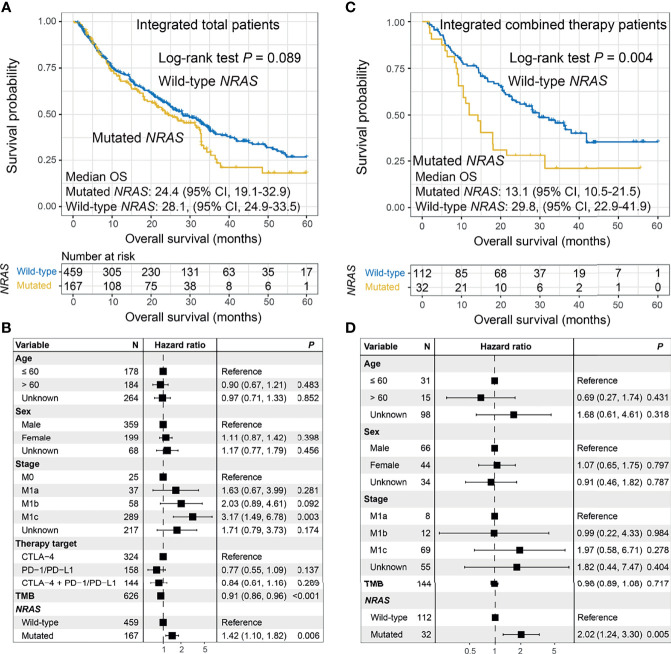
Association between *NRAS* mutations and immune checkpoint inhibitor (ICI) survival. **(A)** Overall survival (OS) curves stratified by *NRAS* mutational status and **(B)** forest plot representation of the connection of *NRAS* mutations with OS outcome in the aggregated melanoma cohort. **(C)** OS curves stratified by *NRAS* mutational status and **(D)** forest plot representation of the association of *NRAS* mutations with ICI outcome in patients who received combined therapy.

Patients with *NRAS* mutations exhibited a trend of worse ICI OS than patients without *NRAS* mutations (median OS: 24.4 [95% CI, 19.1–32.9] vs. 28.1 [95% CI, 24.9–33.5] months; log-rank test *p* = 0.089; **Figure 3A**). This result was more significant in the multivariate Cox model (HR: 1.42, 95% CI: 1.10–1.82, *p* = 0.006; **Figure 3B**). No significant difference was observed between patients with and without *NRAS* mutations in relation to ICI PFS (HR: 1.00, 95% CI: 0.74–1.33, *p* = 0.998; **Figures S10A, B**). The tendencies of decreased ORR (OR: 1.34, 95% CI: 0.89–2.04, *p* = 0.161; **Figure S10C**) and DCR (OR: 1.27, 95% CI: 0.86–1.88, *p* = 0.231; **Figure S10D**) were observed in *NRAS*-mutated tumors. The associations between *NRAS* mutations and ICI OS in the three distinct treatments were also assessed. The results demonstrated that *NRAS* mutations were consistently correlated with immune resistance in combined therapy using the Kaplan–Meier analysis (log-rank test *p* = 0.004; **Figure 3C**) and multivariate Cox model (HR: 2.02, 95% CI: 1.24–3.30, *p* = 0.005; **Figure 3D**), as well as anti-CTLA-4 therapy (log-rank test *p* = 0.132; multivariate Cox HR: 1.65, 95% CI: 1.16–2.36, *p* = 0.005; **Figures S11A, B**). No significant correlation of *NRAS* mutations with anti-PD-1/PD-L1 outcomes was observed (**Figures S11C, D**). Verifiably, *NRAS* mutations were marginally associated with ICI resistance in the combined therapy in the MSKCC cohort (log-rank test *p* = 0.189; multivariate Cox HR: 1.93, 95% CI: 0.90–4.12, *p* = 0.085; **Figures S12A, B**).

*NARS2* mutations were associated with an elevated ORR (OR: 0.15, 95% CI: 0.03–0.57, *p* = 0.008; **Figure S13A**), and a similar tendency was also observed in DCR (OR: 0.31, 95% CI: 0.07–1.13, *p* = 0.096; **Figure S13B**). No differences were detected between the OS curves stratified by *NARS2* status (HR: 1.57, 95% CI: 0.69–3.61, *p* = 0.286; **Figure S13C**). However, a shortened PFS was observed in patients with *NARS2* mutations (HR: 2.52, 95% CI: 1.12–5.68, *p* = 0.033; **Figure S13D**).

*DCC* mutations were correlated with enhanced ORR (OR: 0.62, 95% CI: 0.39–0.98, *p* = 0.041; **Figure S14A**), and a similar tendency was also observed in DCR (OR: 0.69, 95% CI: 0.44–1.08, *p* = 0.102; **Figure S14B**). Survival and Cox regression analyses indicated that patients with *DCC* mutations exhibited the trends of improved OS (HR: 0.80, 95% CI: 0.59–1.10, *p* = 0.167; **Figure S14C**) and PFS (HR: 0.71, 95% CI: 0.49–1.05, *p* = 0.082; **Figure S14D**), although not statistically significant.

### Mutational Signatures Associated With Immune Checkpoint Inhibitor Response or Resistance

**Figure 4:**
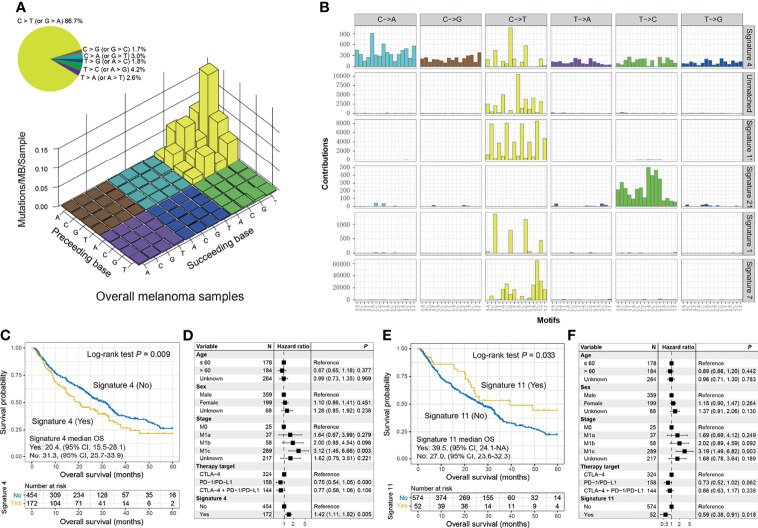
Mutational signatures extracted from the integrated melanoma cohort and their association with immunotherapy prognosis. **(A)** Lego plot representation of mutation patterns in 631 melanoma cases. Single-nucleotide substitutions are divided into 6 categories with 16 surrounding flanking bases. The inset pie chart displays the proportion of 6 mutational patterns. **(B)** The activities of corresponding extracted mutational signatures (i.e., signatures 1, 4, 7, 11, and 21, and unmatched signature). The trinucleotide base substitution types are shown on the x-axes, whereas the y-axes illustrate the contribution percentage of distinct mutation types in each mutational signature. The Kaplan–Meier overall survival (OS) analysis of **(C)** signature 4 and **(E)** signature 11. Multivariate Cox regression models of **(D)** signature 4 and **(F)** signature 11 with age, sex, stage, and therapy type taken into account.

The overall mutational pattern of pooled melanoma patients was dominated by C > T (or G > A) mutations with a mutational proportion of 86.7% (**Figure 4A**). We extracted six mutational signatures from melanoma and subsequently compared them with 30 validated signatures from COSMIC. Finally, signatures 1, 4, 7, 11, and 21 were determined according to the COSMIC nomenclature, and a novel signature (named as the unmatched signature) that did not match the previously annotated mutational signatures was also uncovered (**Figure 4B** and **Figure S15**). The distribution of six mutational signatures in each patient varied, as illustrated in **Table S5** and **Figure S16**. Clock-like signature 1, characterized by C > T mutations at CpG dinucleotides, was associated with age-related accumulation of spontaneous deamination of 5-methylcytosine. Signature 4 is featured by C > A mutations and has been reported to be connected with exposure to tobacco carcinogens (e.g., benzo[*a*]pyrene). Mutational profiles of signatures 7 and 11, which both exhibited mainly C > T substitutions and predominantly existed in melanoma, are likely due to exposure to ultraviolet light and treatment with alkylating agents, respectively. Signature 21, dominated by T > C mutations, is probably linked to microsatellite unstable tumors. The unmatched signature was characterized by C > T mutations.

We observed that the presence of signature 4 was significantly correlated with ICI resistance in OS analysis (median OS: 20.4 [95% CI, 15.5–28.1] vs. 31.3 [95% CI, 25.7–33.9] months; log-rank test *p* = 0.009; **Figure 4C**) and multivariate-adjusted model (HR: 1.42, 95% CI: 1.11–1.82, *p* = 0.005; **Figure 4D**). A tendency of worse PFS outcome was also observed in patients with signature 4 (log-rank test *p* = 0.196; multivariate Cox *p* = 0.152; **Figure S17A**). Consistently, decreased ORR (20.6% vs. 34.9%; Fisher’s exact test *p* < 0.001; multivariate logistic *p* = 0.001; **Figure S17B**) and DCR (33.9% vs. 49.8%; Fisher’s exact test *p* < 0.001; multivariate logistic *p* < 0.001; **Figure S17C**) were associated with the tumors with signature 4. We also compared the genomic and microenvironmental features of patients with and without signature 4. A decreased TMB was observed in patients with signature 4 (Wilcoxon’s rank-sum text *p* < 0.001; multivariate logistic OR: 0.10, 95% CI: 0.06–0.16, *p* < 0.001; **Figures S18A, B**). In addition, the lower infiltration of M1 macrophages (Wilcoxon’s rank-sum text *p* = 0.007; **Figure S18C**) and higher infiltration of M2 macrophages (Wilcoxon’s rank-sum text *p* = 0.045; **Figure S18C**) may be another reason for the ICI resistance of patients with signature 4.

Conversely, the presence of signature 11 was linked to improved ICI OS in survival analysis (median OS: 39.5 [95% CI, 24.1–NA] vs. 27.0 [95% CI, 23.6–32.3]; log-rank test *p* = 0.033; **Figure 4E**) and multivariate Cox regression model (HR: 0.59, 95% CI: 0.38–0.91, *p* = 0.018; **Figure 4F**). Improved ICI OS was also observed in patients with the unmatched signature (HR: 0.59, 95% CI: 0.39–0.90, *p* = 0.014; **Figures S19A, B**). We also treated the above three signatures as continuous variables to conduct a multivariate Cox analysis. The associations of signature 4 (HR: 2.04, 95% CI: 1.26–3.29, *p* = 0.003; **Figure S20A**), signature 11 (HR: 0.47, 95% CI: 0.23–0.97, *p* = 0.041; **Figure S20B**), and unmatched signature (HR: 0.35, 95% CI: 0.12–1.01, *p* = 0.052; **Figure S20C**) with ICI OS were still present.

### Potential Molecular Subtypes Contributed to Immune Checkpoint Inhibitor Overall Survival

We could detect latent molecular subtypes based on the activities of extracted mutational signatures. Consensus clustering analysis was performed with cluster numbers ranging from 2 to 10. We observed that the preferable clustering consensus was exhibited when clustering numbers were selected as three or five (**Figure S21A**). More subtle subtypes could be virtually microdissected with an increase in clustering numbers as shown in the cluster tracking plot (**Figure S21B**). Therefore, we selected the clusters as five (i.e., C1, C2, C3, C4, and C5) to explore their association with ICI OS. The plots of the cluster consensus and consensus matrix are separately illustrated in **Figures S21C, D**.

**Figure 5:**
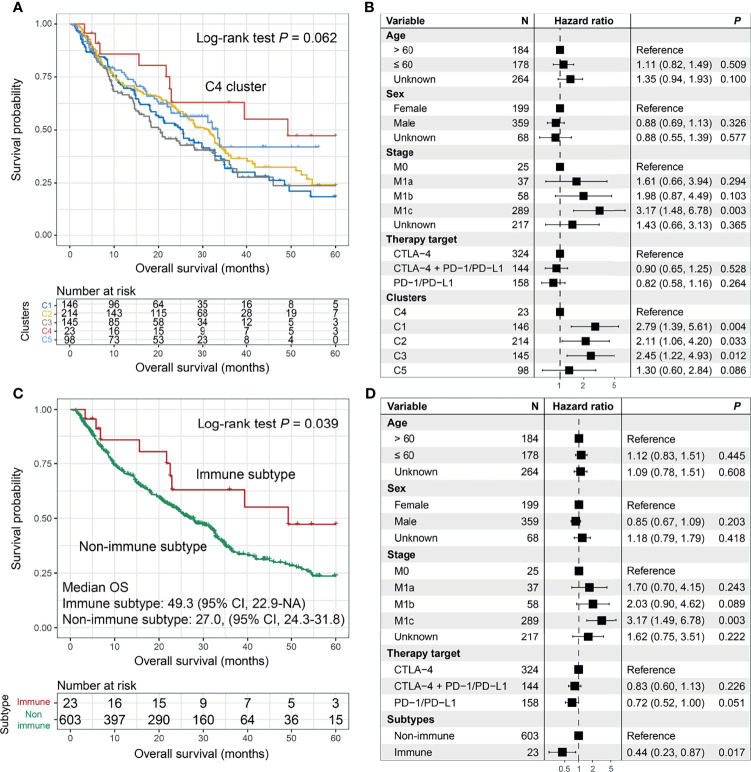
The prediction roles of the identified melanoma immune subtype for immune checkpoint inhibitor (ICI) survival. **(A)** Kaplan–Meier survival analysis and **(B)** forest plot illustration of 5 clusters derived from the consensus clustering. Prognostic significances of the immune subtype vs. non-immune subtype under **(C)** Kaplan–Meier overall survival (OS) analysis and **(D)** multivariate Cox model with confounding variables taken into consideration.

The Kaplan–Meier analysis suggested that patients from the C4 cluster (23 of 626 patients [3.7%]) could achieve the best ICI OS as compared with the other four clusters (log-rank test *p* = 0.062; **Figure 5A**). In the multivariate Cox model, we treated the C4 cluster as the reference subgroup and observed that the other four clusters exhibited worse ICI OS (*p* = 0.004, 0.033, 0.012, and 0.086; **Figure 5B**). In this study, we termed the C4 cluster as “Immune subtype” and the rest as “Non-immune subtype”. The improved ICI OS of the immune subtype was still observed when compared with the non-immune subtype in univariate analysis (median OS: 49.3 [95% CI, 22.9–NA] vs. 27.0 [95% CI, 24.3–31.8] months; log-rank test *p* = 0.039; **Figure 5C**) and multivariate Cox model (HR: 0.44, 95% CI: 0.23–0.87, *p* = 0.017; **Figure 5D**).

### The Combined Biomarker Predictive of Immune Checkpoint Inhibitor Overall Survival

Considering the predictive implications of *COL3A1* mutations and signature 4, we integrated *COL3A1* mutations and lack of mutational signature 4 as a combined biomarker to evaluate the improved ICI OS (**Table S6**). Patients with the combined marker harbored a significantly better ICI OS than patients without the combined marker (median OS: 45.0 [95% CI, 34.5–NA] vs. 24.9 [95% CI, 21.5–28.2] months; log-rank test *p* < 0.001; **Figure S22A**). The association remained still significant even after adjusting for the confounding factors in the multivariate Cox regression model (HR: 0.60, 95% CI: 0.42–0.87, *p* = 0.007; **Figure S22B**). The presence of the combined marker was also associated with improved ICI PFS according to the Kaplan–Meier analysis (median PFS: 11.43 [95% CI, 6.23–NA] vs. 4.47 [95% CI, 3.50–6.03] months; log-rank test *p* = 0.013; **Figure S22C**) and multivariate Cox model (HR: 0.62, 95% CI: 0.41–0.94, *p* = 0.026; **Figure S22D**). Consistently, an elevated ORR was observed in patients with the combined marker in the univariate analysis (45.2% vs. 28.1%; Fisher’s exact test *p* = 0.001; **Figure S23A**) and multivariate logistic regression (OR: 0.67, 95% CI: 0.41–1.11, *p* = 0.043; **Figure S23B**). A similar association between the combined marker and DCR was also found by employing univariate (60.6% vs. 42.5%; Fisher’s exact test *p* < 0.001; **Figure S23C**) and multivariate analysis (OR: 0.59, 95% CI: 0.36–0.97, *p* = 0.035; **Figure S23D**).

## Discussion

Immune checkpoint-based treatments have revolutionized therapeutic strategies for melanoma. In this study, we comprehensively explored the mutational profiles of 631 melanoma patients treated with ICI agents. We identified four novel SMGs that were previously not recognized to be associated with ICI response/resistance. We further annotated three mutational signatures with respect to ICI efficacy. In addition, a latent immune subtype was demonstrated to be linked to improved ICI outcomes.

Mutations in single genes, such as *MUC16* (22), *POLE* (19), and *PBRM1* (20), exhibited vital effects on the prediction of tumor prognoses or immunotherapeutic outcomes. Our results showed that mutations in the newly identified *COL3A1* SMG were linked with improved ICI response and survival. Subsequently, genomic and immunologic analyses explained that enhanced TMB and NB, and a hot immune microenvironment characterized patients with *COL3A1* mutations. Indeed, *COL3A1* also participates in immune response regulation at the gene expression level (50, 51), and further studies are needed to explore the link between *COL3A1* mutations and protein expression in immunotherapy. In this study, we also observed that melanoma patients with and without *COL3A1* mutations exhibited a survival difference in the setting of anti-CTLA-4 therapy, but not in the anti-PD-1/PD-L1 therapy. This may be attributed to the following three reasons: 1) the distinct interactions of *COL3A1* mutations with CTLA-4 and PD-1/PD-L1, for example, synergistic and antagonistic roles; 2) the tumor microenvironment may be distinctly influenced by the two ICI treatments, which would generate differential immunogenicity in patients with *COL3A1* mutations; and 3) the sample size used for the two ICI types (324 vs. 158) may also be a potential reason for the distinct survival differences.

The association of *NRAS* mutations with ICI efficacy was only reported by Johnson et al. (52), and their observations indicated trends of improved ICI OS (19.5 vs. 15.2 months) and PFS (4.1 vs. 2.9 months) for patients with *NRAS* mutations, although the results were not statistically significant (log-rank test *p* = 0.51 and 0.08, respectively). Conversely, our study revealed that *NRAS* mutations were linked with inferior ICI OS in the aggregated cohort (multivariate Cox HR: 1.42, *p* = 0.006), and this result was also obtained in both combined therapy (multivariate Cox HR: 2.02, *p* = 0.005) and anti-CTLA-4 cohort (multivariate Cox HR: 1.65, *p* = 0.005). Furthermore, a similar tendency of poorer OS was also observed in patients with *NRAS* mutations who received combined therapy in the MSKCC cohort (multivariate Cox HR: 1.93, *p* = 0.085). The inconsistent results may be attributed to the following two reasons: 1) the sample sizes used, 631 samples of our study vs. 229 of Johnson et al. study; 2) in the multivariate-adjusted analysis, we performed multivariate Cox regression models adjusting for confounding factors (e.g., age, sex, stage, therapy types, and TMB); however, no adjusted analyses were applied in the Johnson et al. study. On the other hand, *NRAS*-mutated patients had a higher TMB, although ICI resistance developed in these patients. This indicates that a high TMB may be a spurious participant in ICI response. Similar results were also reported by Marinelli et al. (53); that is, *KEAP1*-driven co-mutations were associated with unresponsiveness to immunotherapy, although an elevated TMB was observed in this subset. The cold microenvironment and other immunological factors present in these patients may significantly contribute to immunotherapy efficacy.

*NARS2* mutations were linked to elevated ORR. However, worse PFS was observed in *NARS2*-mutated patients. These results indicate that *NARS2* mutations may be a favorable indicator for shorter treatment responses; however, they may play a negative role in disease prognosis.

Smoking-related mutational signature 4, which commonly occurs in lung, head, and neck, and esophageal cancers, was also detected in the pooled melanoma cohort. Lung cancer patients harboring this mutational signature have been demonstrated to show a higher response to ICI treatment (12). However, our study indicated that the smoking signature was associated with ICI resistance in melanoma patients, and distinct tumor types may generate inconsistent results. Findings from a recent study (54) revealed that melanoma patients with cigarette smoking behavior exhibited inferior melanoma-specific survival, which was due to smoking-associated decreased immune infiltration. In our study, the smoking signature was also correlated with a weaker immune microenvironment *via* the regulation of M1 and M2 macrophages. Overall, smoking and its relevant traits may influence immune responses and thus determine the prognosis and immunotherapeutic efficacy in melanoma.

In our study, melanoma patients with alkylating agent exposure-related mutational signature 11 showed prolonged survival as compared with those without such signature. Consistently, patients who received ICI agents were more likely to experience an enhanced ORR if their tumors had this alkylating agent signature in melanoma (27). We also identified a mutational signature that featured C > T substitutions, which was associated with improved survival following ICI treatment. The discovery of this novel mutational signature would further enrich COSMIC data and provide implications for immunotherapy.

The immune molecular subtypes were commonly identified based on immunologic and microenvironment characterizations derived from mixed gene expression profiles. However, currently, a majority of immunotherapy studies have mainly focused on the somatic mutation level, and fewer included gene expression data. In this integrated analysis, only three of eight cohorts had mRNA sequencing data; thus, it may be inappropriate to conduct molecular subtyping by employing mRNA expression data with the limited coverage of melanoma patients. The utilization of activities of mutational signatures extracted from tumor samples is a good choice to determine immanent subclasses in patients with only or mainly mutation data. We detected five clusters with distinct survival outcomes using the six mutational signatures. One cluster with the best ICI prognosis was termed the immune subtype in this study. Interestingly, we observed that patients of the immune subtype were a subset of patients with a lack of signature 4 and the presence of signature 11 (**Table S6**), further verifying the favorable prognostic outcome of this immune subtype.

Based on the findings of this study, prospective clinical trials should be performed to confirm the potential implications of *COL3A1* mutations, mutational signature 4, the identified immune subtype, and other immunotherapy determinants in melanoma and other cancer types, which will provide more clues for guiding clinical practice and individualized treatment. However, there are several limitations to this research. First, the integrated melanoma cohort was derived from multiple distinct cohorts, which may produce deviations in the data processing. Second, transcriptomic data were obtained from only three of the eight included studies, which may not fully elucidate the potential mechanisms of the determinants. Finally, the associations of the identified gene mutations with immunological features remained at a theoretical level and need to be experimentally validated.

Overall, our study integrated 631 ICI-treated melanoma patients and uncovered several clinically related ICI determinants, which provide helpful biomarkers for melanoma immunotherapy prediction.

## Data Availability Statement

Publicly available datasets were analyzed in this study. This data can be found here: https://xenabrowser.net/hub/ https://www.tcia.at/home.

## Ethics Statement

All studies have been reviewed and approved by the Institutional Research Board.

## Author Contributions

QW designed this study. QW, SW, WZ, and YK developed the methodology and acquired the related data. QW, WZ, YK, YL, FS, JL, and CS performed the data analysis and interpretation. WZ, YK, and QW drafted and revised the manuscript. QW supervised this study. All authors read and approved the final manuscript.

## Funding

This study was supported by the Shandong Provincial Youth Innovation Team Development Plan of Colleges and Universities (No. 2019-6-156, Lu-Jiao), National Natural Science Foundation of China (Nos. 81872719 and 81803337), Provincial Natural Science Foundation of Shandong Province (No. ZR201807090257), and National Bureau of Statistics Foundation Project (No. 2018LY79).

## Conflict of Interest

The authors declare that the research was conducted in the absence of any commercial or financial relationships that could be construed as a potential conflict of interest.

## Publisher’s Note

All claims expressed in this article are solely those of the authors and do not necessarily represent those of their affiliated organizations, or those of the publisher, the editors and the reviewers. Any product that may be evaluated in this article, or claim that may be made by its manufacturer, is not guaranteed or endorsed by the publisher.
