# Supplementary material for: Novel Molecular Determinants of Response or Resistance to Immune Checkpoint Inhibitor Therapies in Melanoma
Source: Front Immunol. 2022 Jan 11;12:798474. doi: 10.3389/fimmu.2021.798474 (PMC8787219; doi:10.3389/fimmu.2021.798474)
Supplement: Supplementary file 1 [file DataSheet_1.docx]

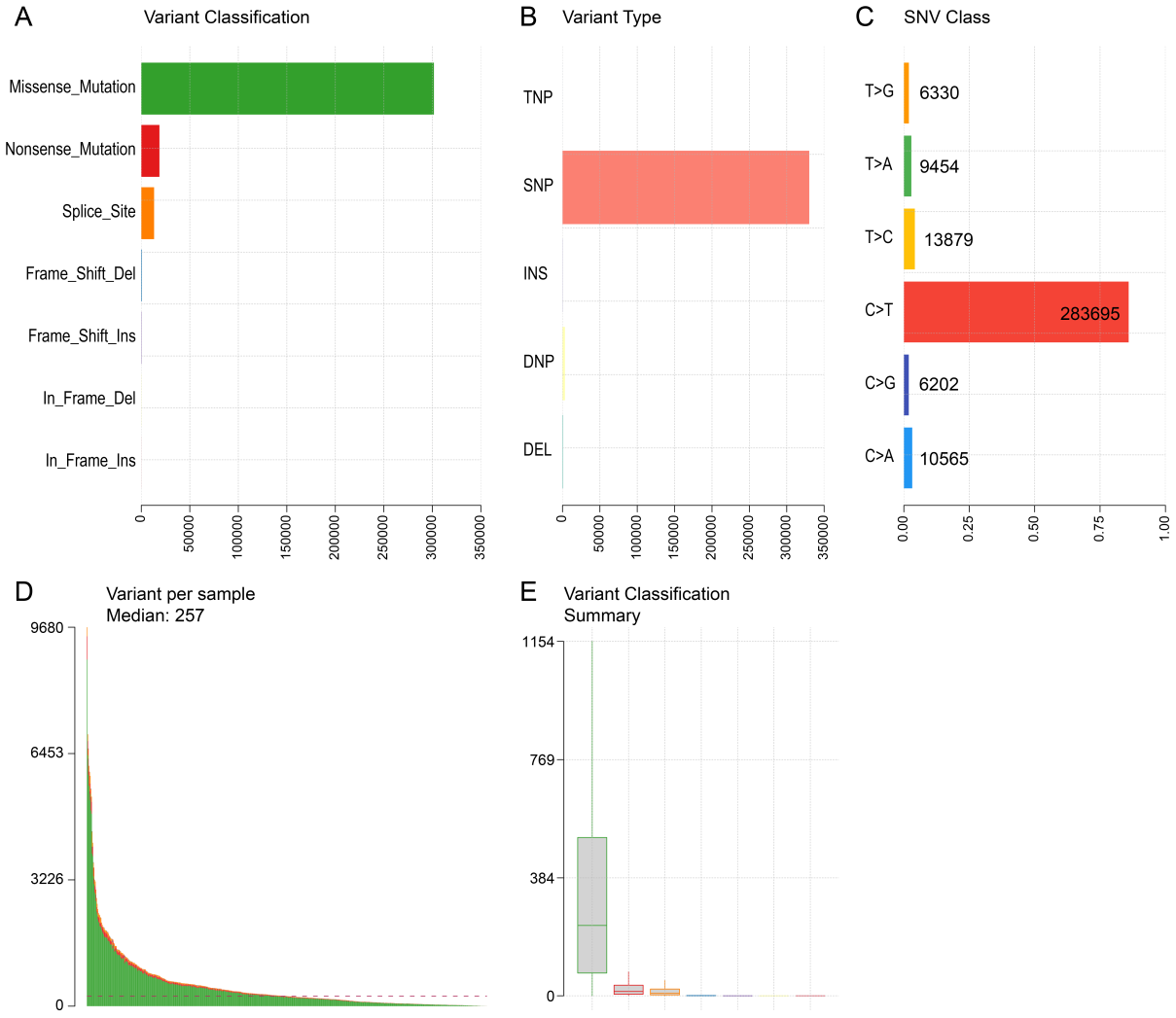
**Supplementary Figures**

Figure S1. Overview of somatic mutational profiles of the aggregated melanoma cohort. Mainly included (A) variant classification, (B) variant type, (C) SNV class, (D) variants per sample, and (E) summary of variant classification.


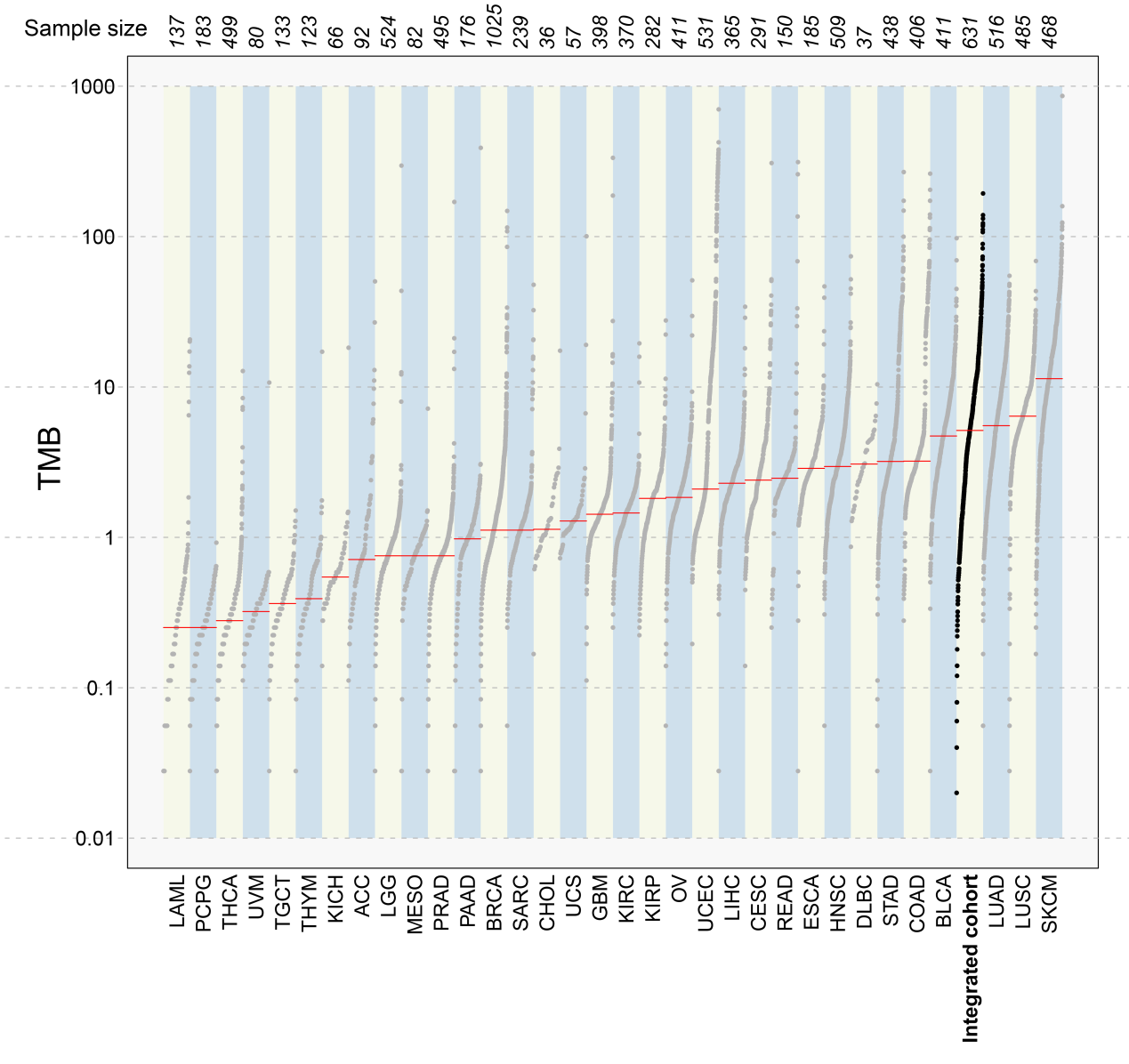


Figure S2. The distribution of TMB across 33 cancer types in TCGA and our aggregated melanoma cohort.


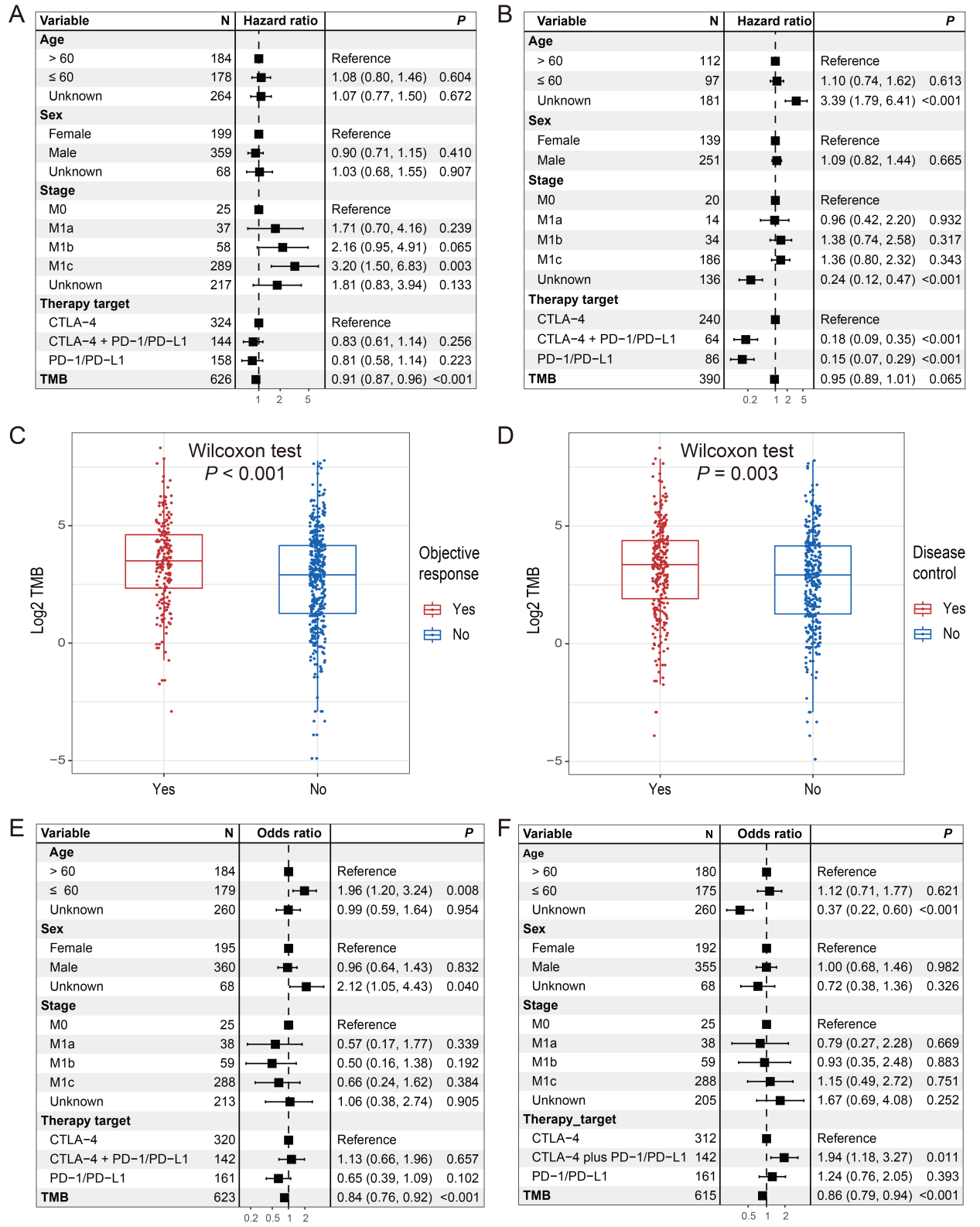


Figure S3. Association of TMB with ICI efficacy. Multivariate Cox regression models of TMB with age, sex, stage, and therapy target taken into account in relation to (A) OS and (B) PFS. Distinct TMB stratified by (C) objective response status and (D) disease control status. Multivariate Logistic regression models adjusted confounding factors were conducted to explore the associations of TMB with (E) objective response and (F) disease control.


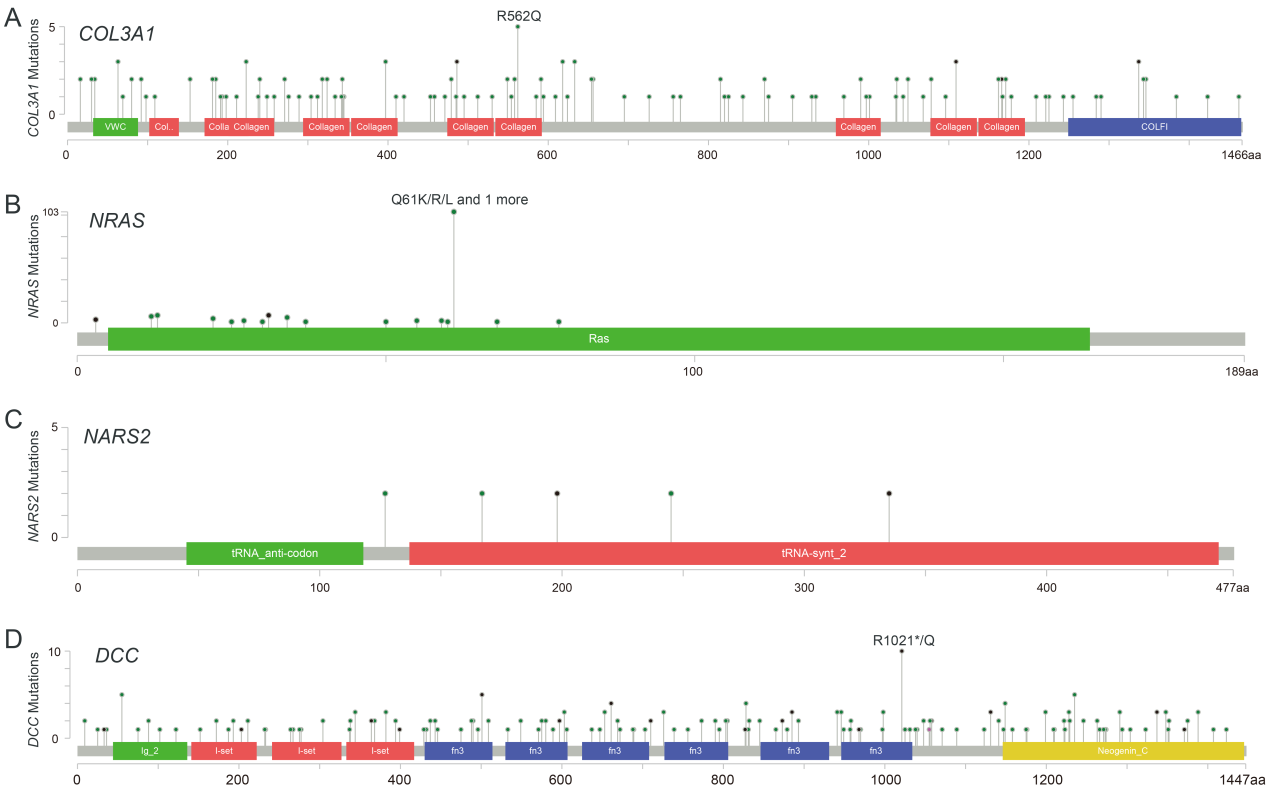


Figure S4. Lollipop charts depiction of detailed mutational information of 4 SMGs, including (A) *COL3A1*, (B) *NRAS*, (C) *NARS2*, and (D) *DCC*.


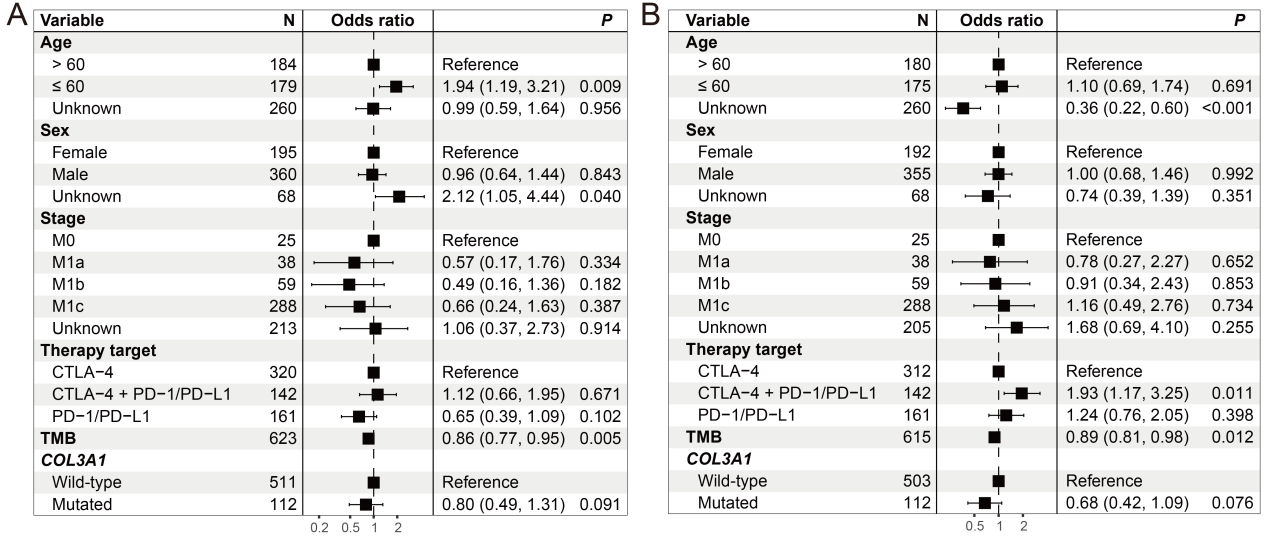


Figure S5. Multivariate Logistic regression analysis of the connections of *COL3A1* mutations with (A) ORR and (B) DCR.


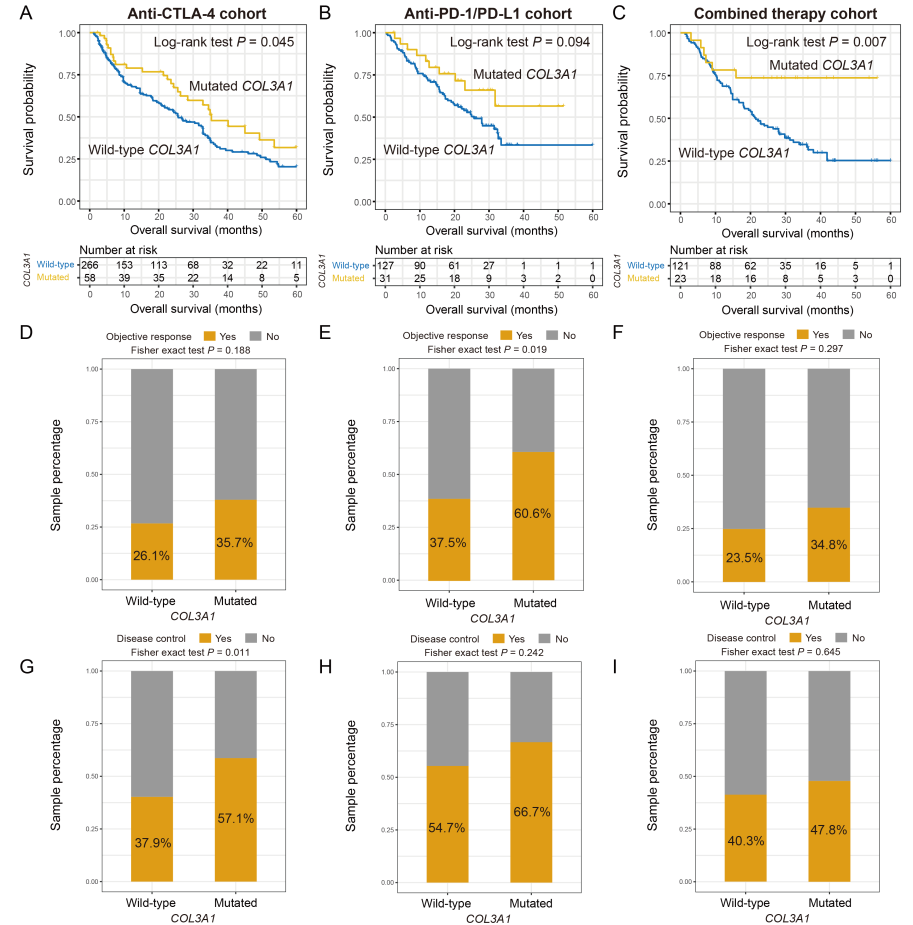
Figure S6. (A, B, C) Kaplan-Meier survival curves, (D, E, F) ORR, and (G, H, I) DCR classified by *COL3A1* mutational status in melanoma patients separately received anti-CTLA-4, anti-PD-1/PD-L1, and combined treatments.


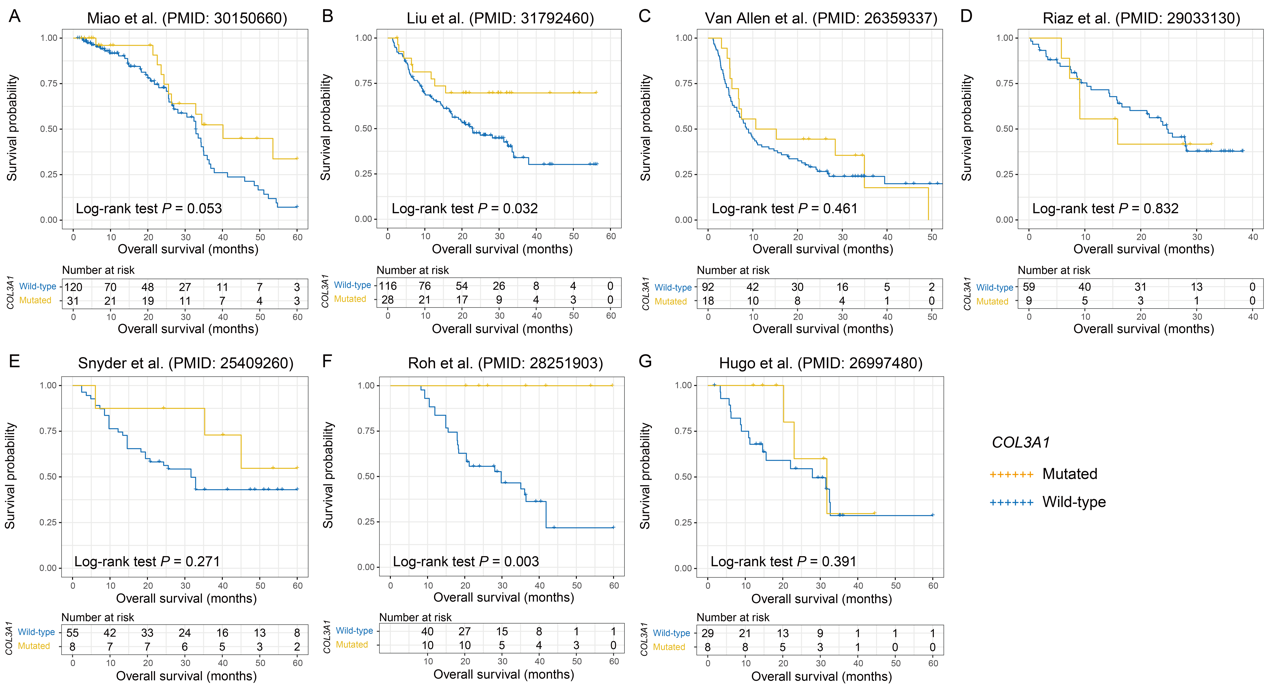


Figure S7. Kaplan-Meier survival analyses of *COL3A1* mutations in 7 individual immunotherapy cohorts, containing studies of (A) Miao *et al.*, (B) Liu *et al.*, (C) Van Allen *et al.*, (D) Riaz *et al.*, (E) Snyder *et al.*, (F) Roh *et al.*, and (G) Hugo *et al.*


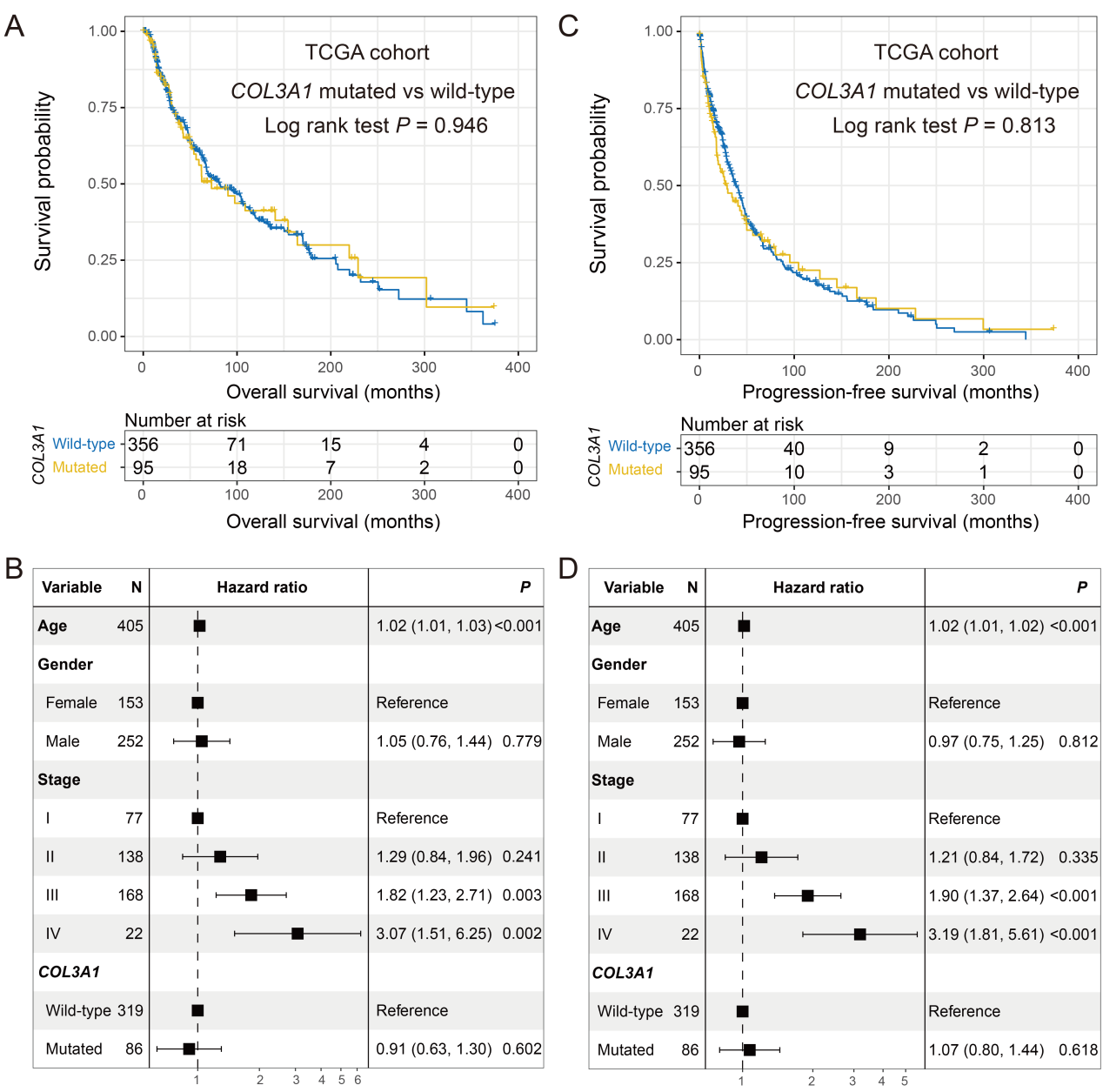


Figure S8. The prognostic significance of *COL3A1* mutations in TCGA cohort. Univariate survival analysis and multivariate Cox regression model were performed to calculate the links of *COL3A1* mutations with (A, B) OS and (C, D) PFS.


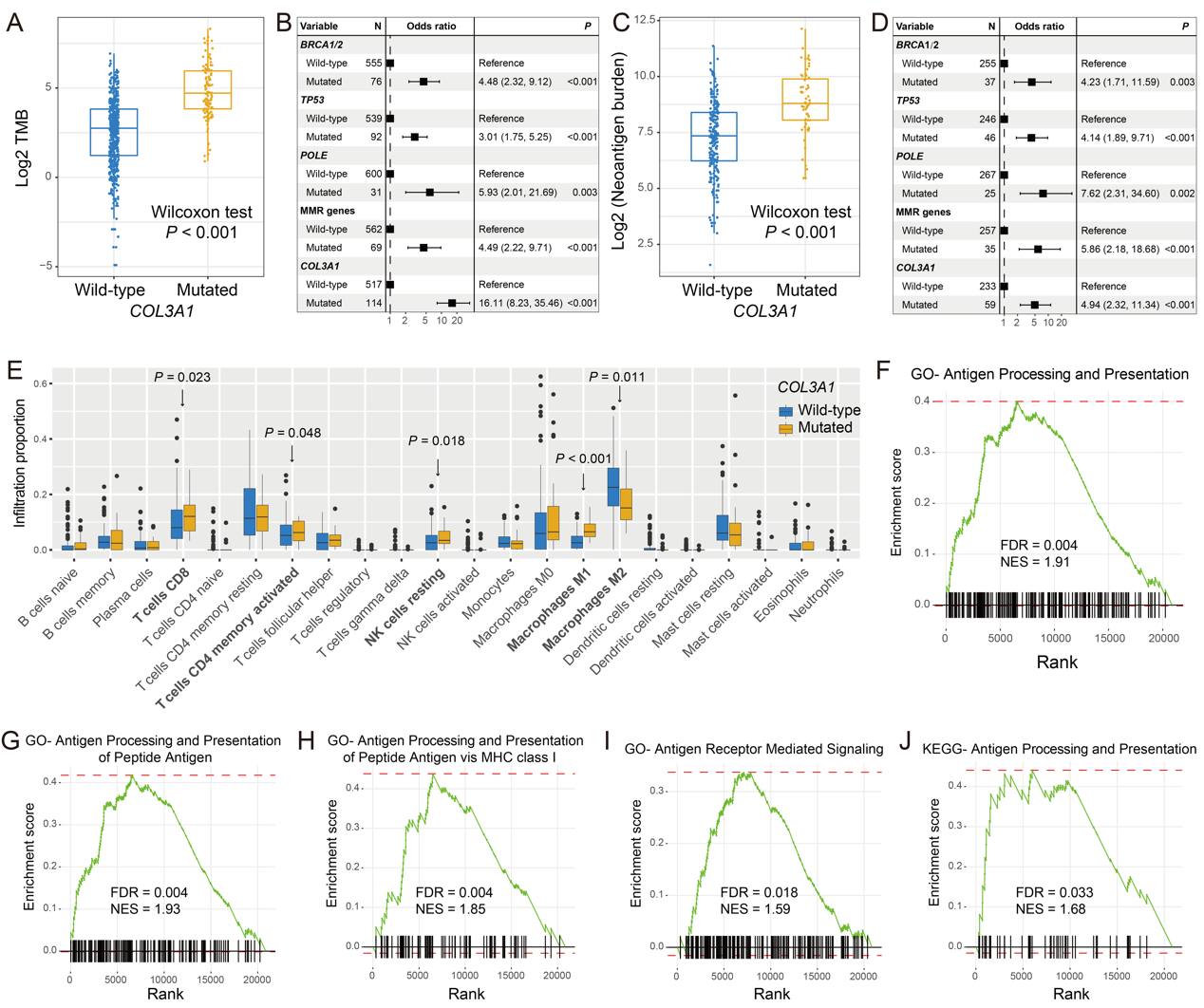


Figure S9. Genomic features and tumor microenvironment with respect to *COL3A1* mutations. (A, B) The association of *COL3A1* mutations with TMB. (C, D) The association of *COL3A1* mutations with NB. (E) Distinct infiltration of 22 immune cells based on *COL3A1* mutational status. (F-J) Antigen processing and presentation relevant signaling pathways enriched by GSEA method.


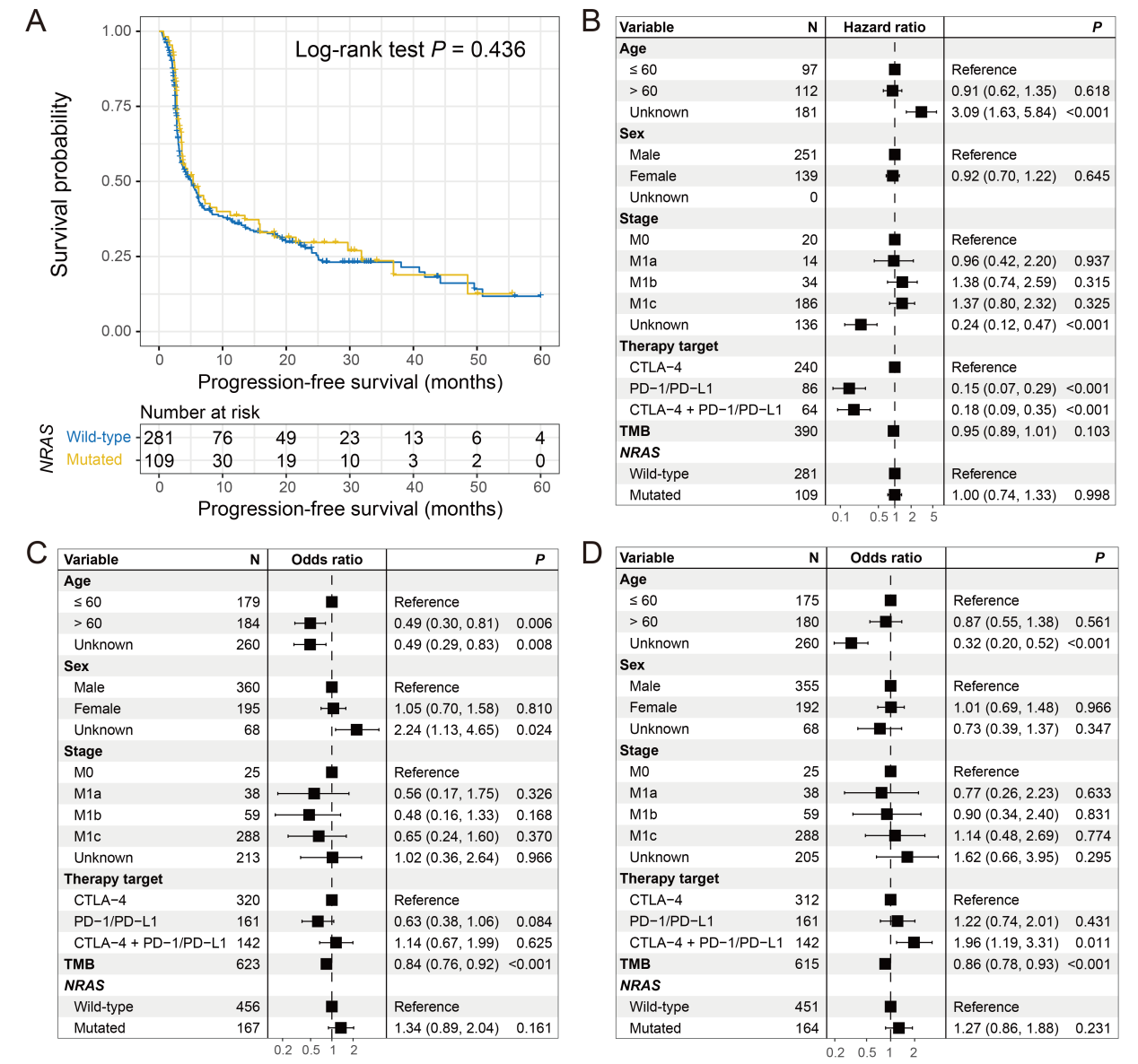


Figure S10. *NRAS* mutations association with ICI PFS outcome under (A) Kaplan-Meier survival analysis and (B) multivariate Cox regression model. Forest plots representation of associations of *NRAS* mutations with (C) ORR and (D) DCR.


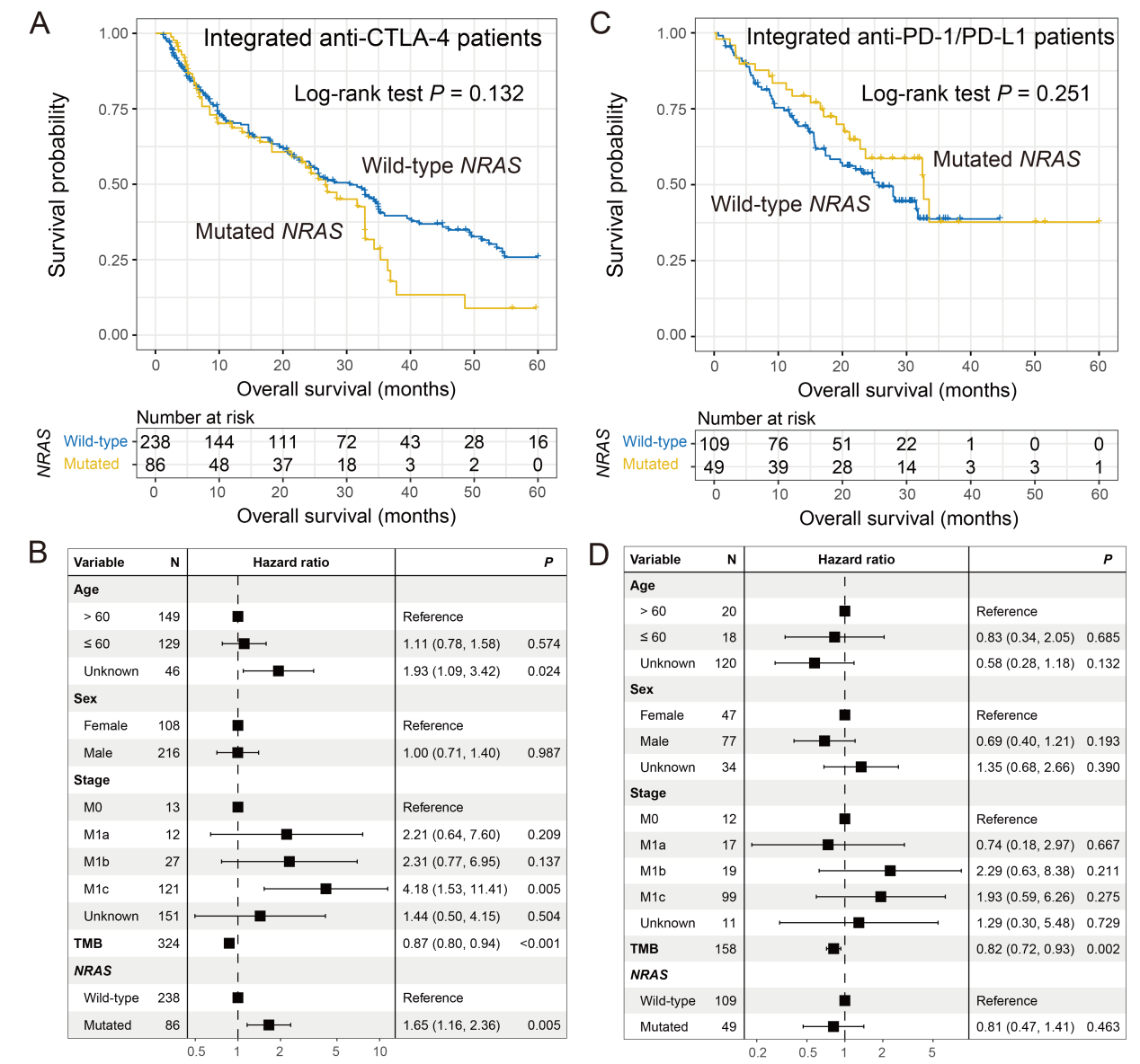


Figure S11. Kaplan-Meier survival and multivariate Cox regression analyses were employed to assess the links between *NRAS* mutations and ICI OS outcome in patients treated with (A, B) anti-CTLA-4 agents and (C, D) anti-PD-1/PD-L1 agents.


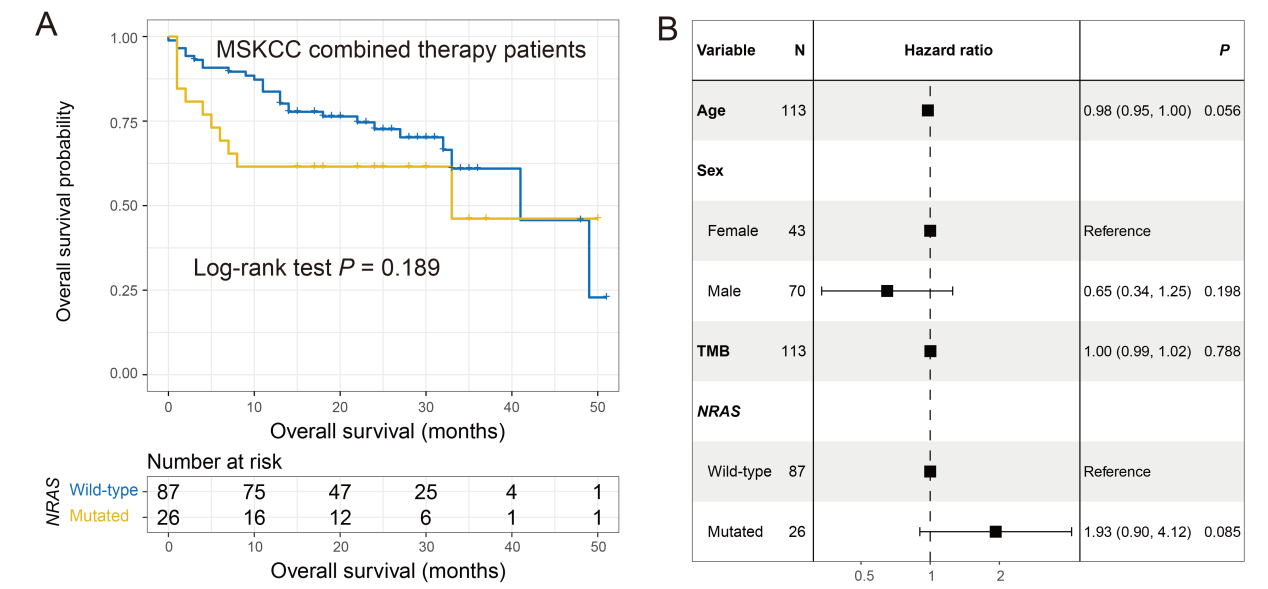


Figure S12. *NRAS* mutations association with ICI survival in the setting of combined therapy in MSKCC cohort. (A) Kaplan-Meier survival and (B) multivariate Cox regression analyses were performed to evaluate the associations between *NRAS* mutations and ICI OS outcome.


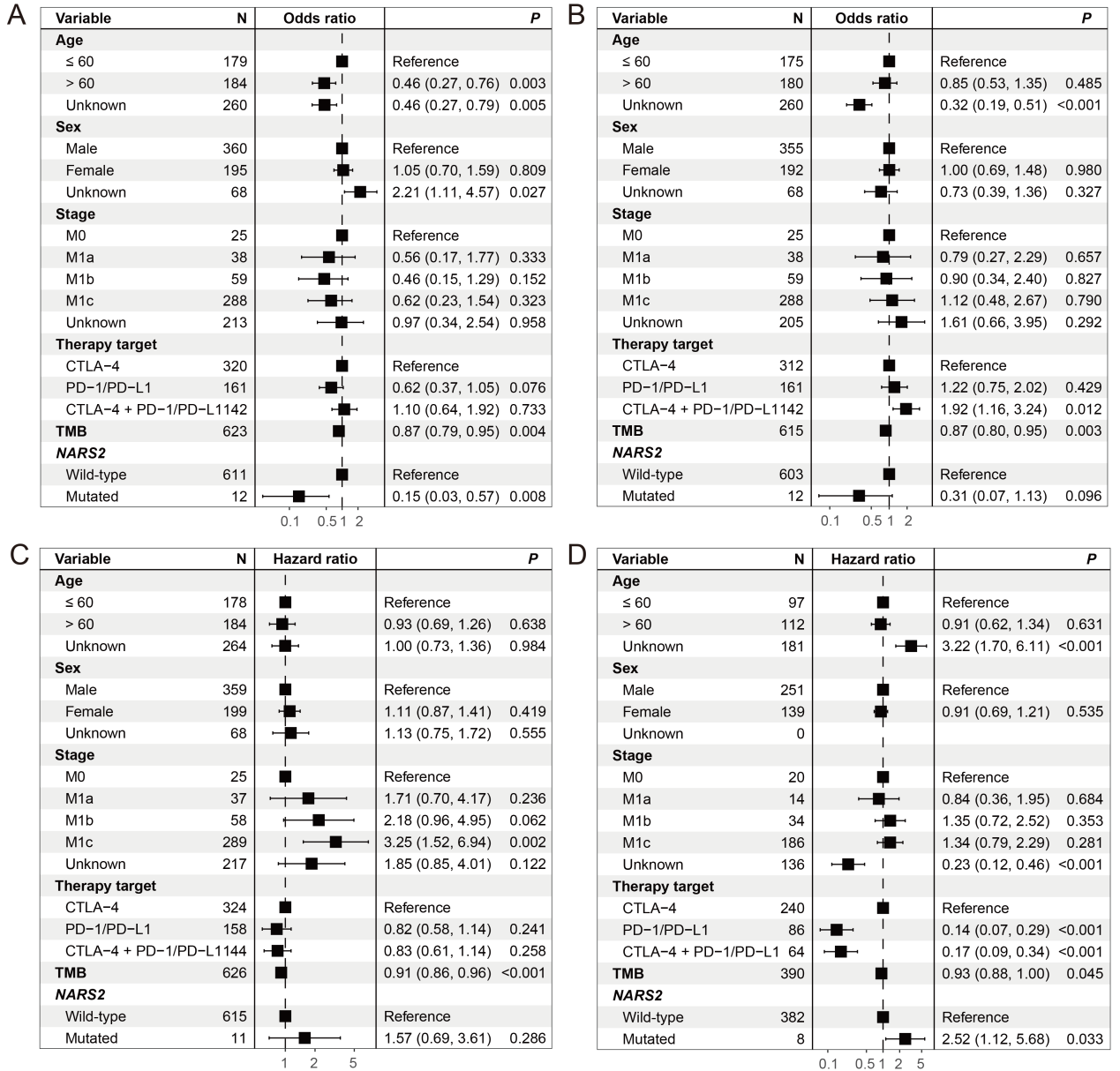


Figure S13. The correlation of *NARS2* mutations with ICI efficacy. Multivariate Logistic regression analyses of associations of *NARS2* mutations with (A) ORR and (B) DCR. Multivariate Cox regression models of associations of *NARS2* mutations with (C) OS and (D) PFS.


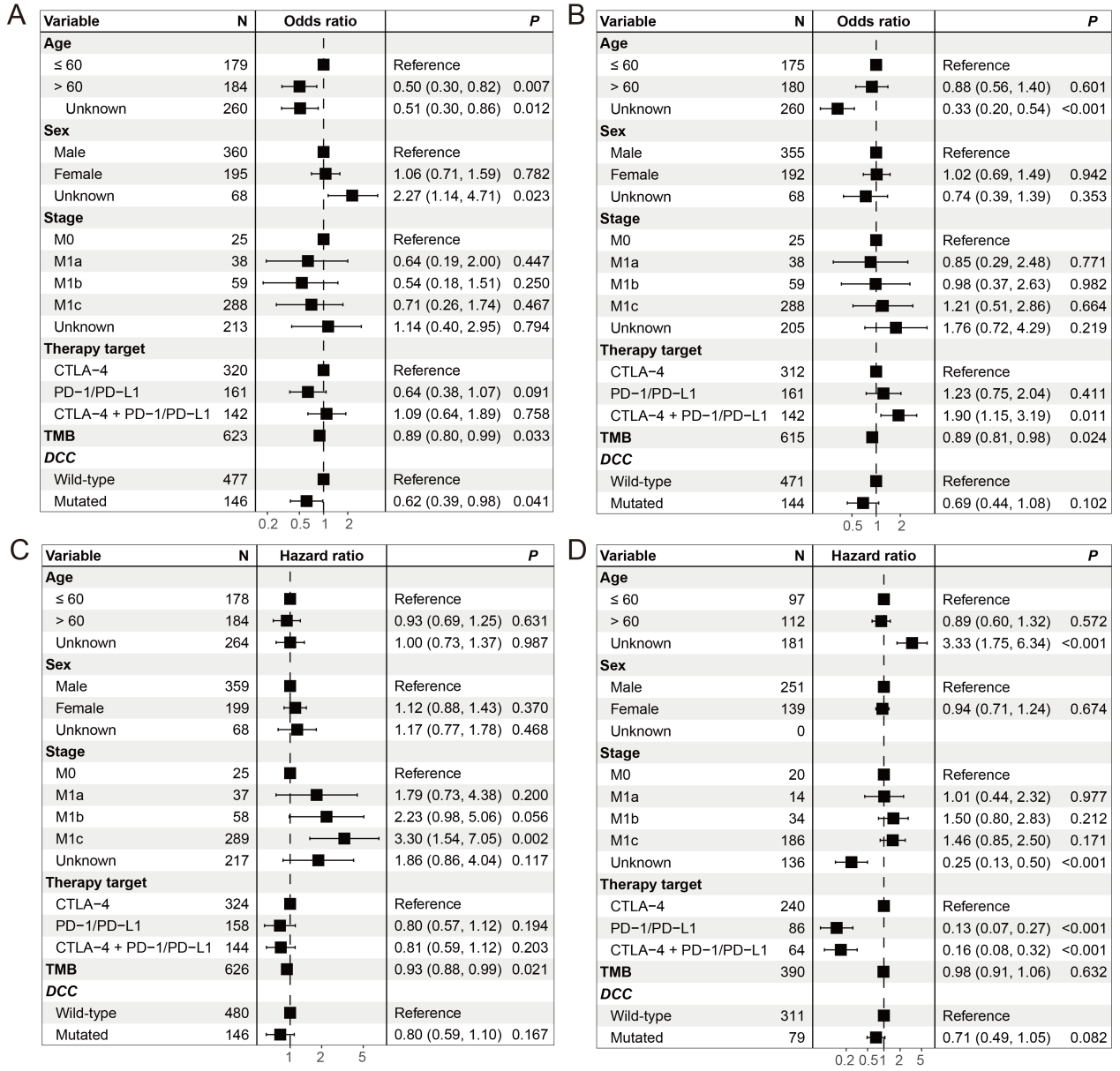


Figure S14. The association of *DCC* mutations with ICI efficacy. Multivariate Logistic regression analyses of associations of *DCC* mutations with (A) ORR and (B) DCR. Multivariate Cox regression models of associations of *DCC* mutations with (C) OS and (D) PFS.


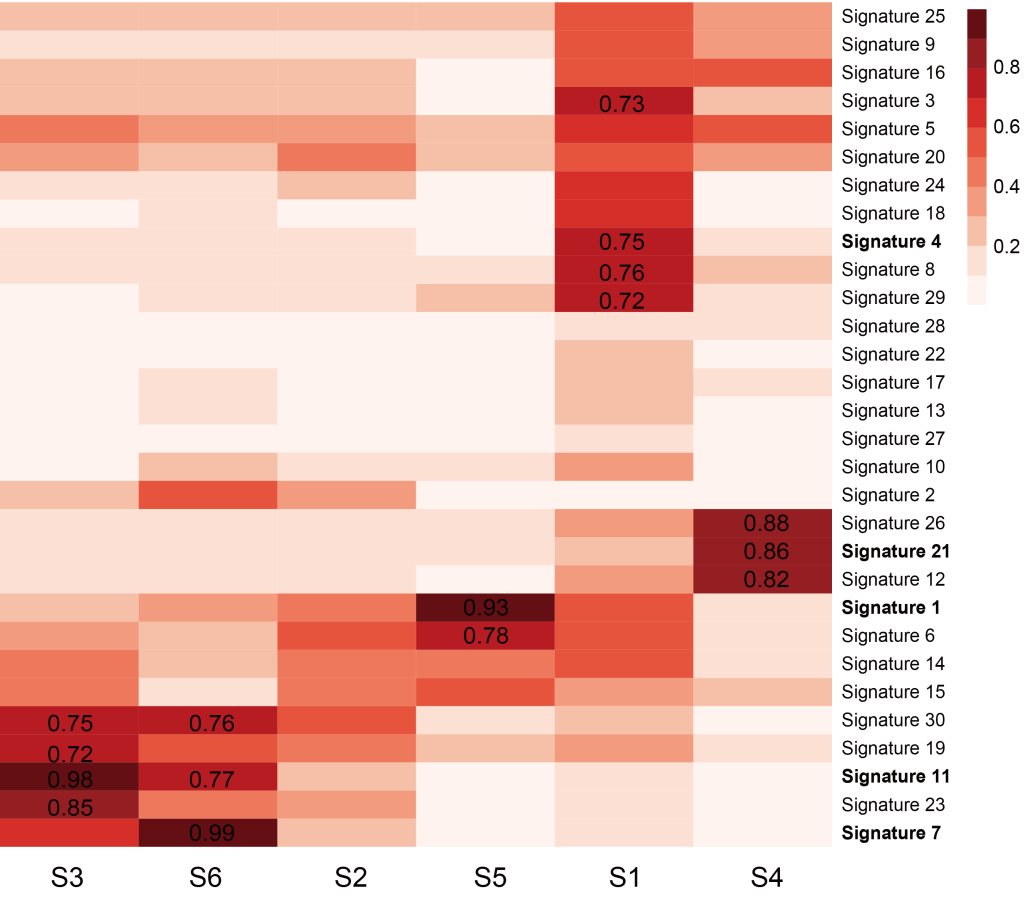
Figure S15. The comparison between extracted 6 mutational signatures and 30 well-annotated signatures in the COSMIC database based on the cosine similarity.


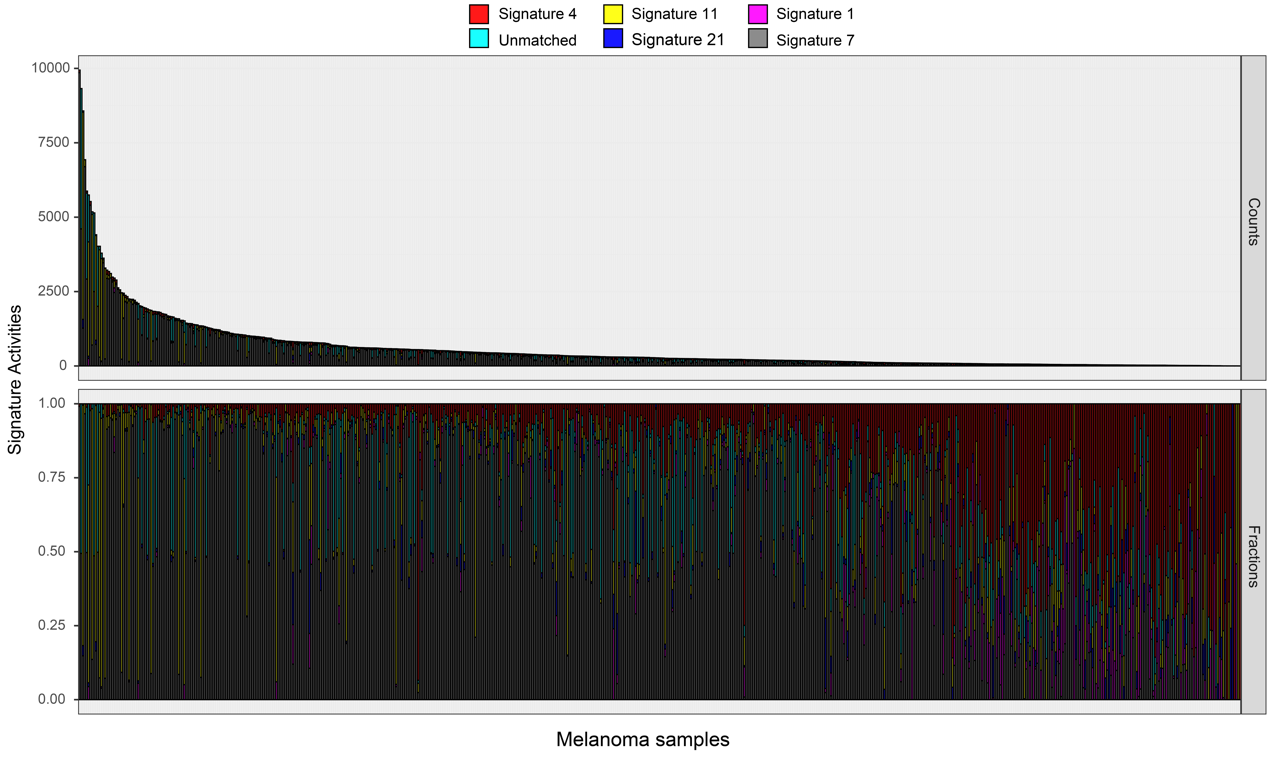


Figure S16. Count and fraction distribution of 6 mutational signatures across all analyzed melanoma patients.


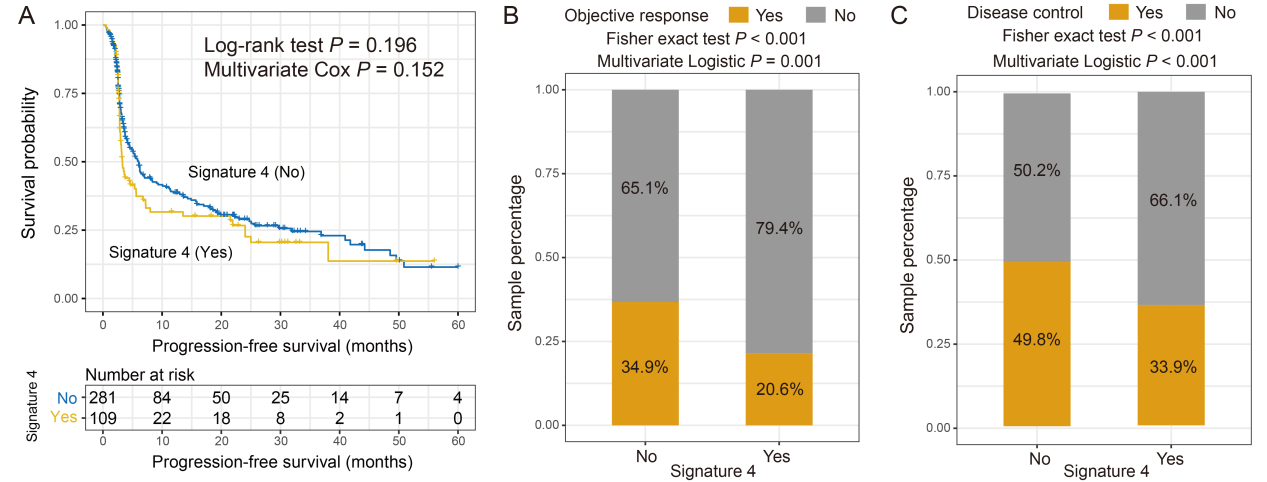


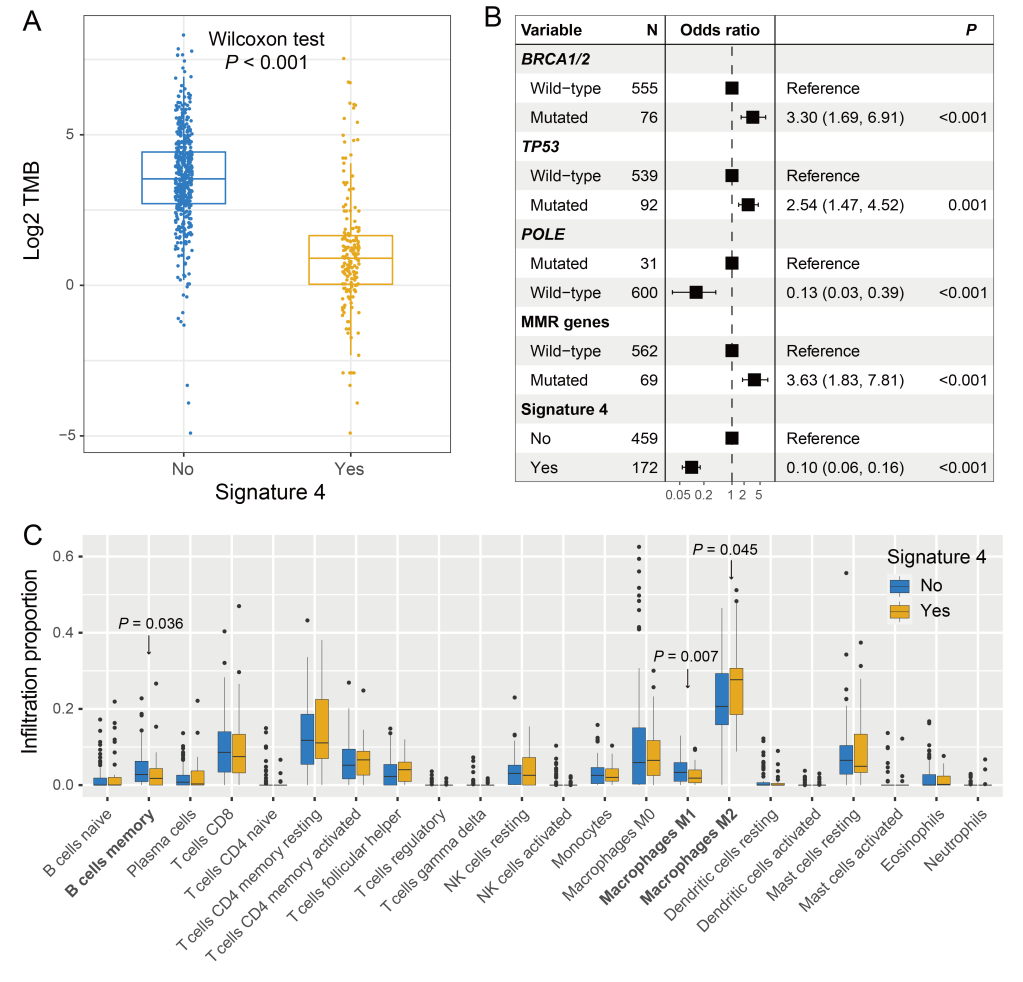
Figure S17. Presence of signature 4 association with (A) ICI PFS outcome, (B) ORR, and (C) DCR by using univariate analysis and multivariate adjusted regression model.

Figure S18. Genomic features and lymphocytes infiltration with respect to the mutational signature 4. (A) Boxplot representation of TMB distribution in patients with and without signature 4. (B) Multivariate Logistic regression model with mutations in genomic maintenance genes taken into account was preformed to assess the association between the presence of signature 4 and TMB. (C) Distinct lymphocytes abundance in patients with signature 4 vs. patients without signature 4.


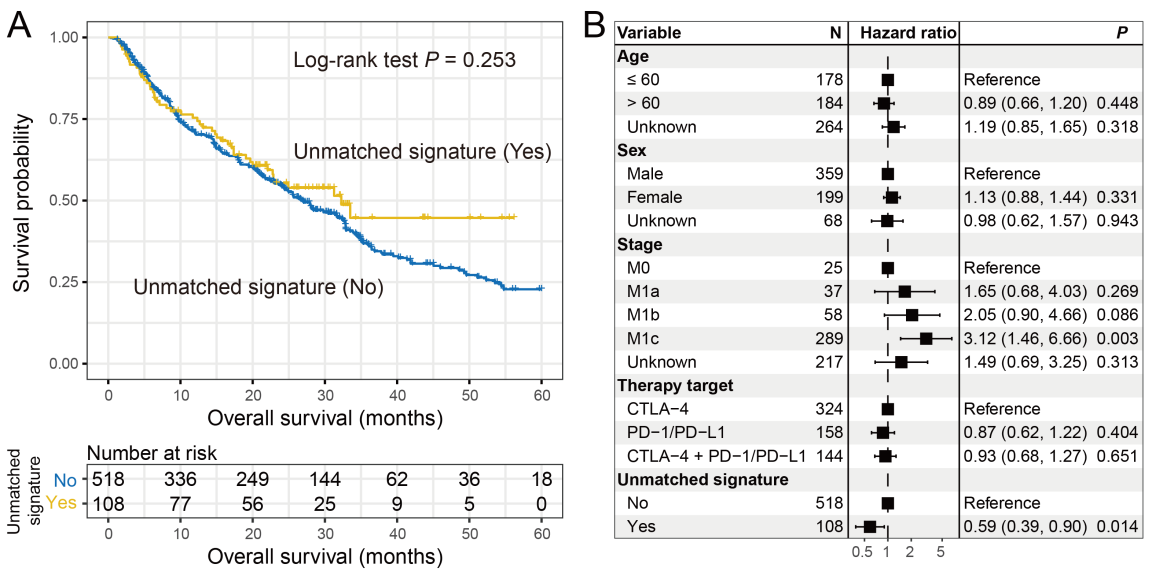
Figure S19. Predictions roles of the novel unmatched mutational signature for ICI OS outcome with (A) Kaplan-Meier survival analysis and (B) multivariate Cox regression model adjusted confounders.


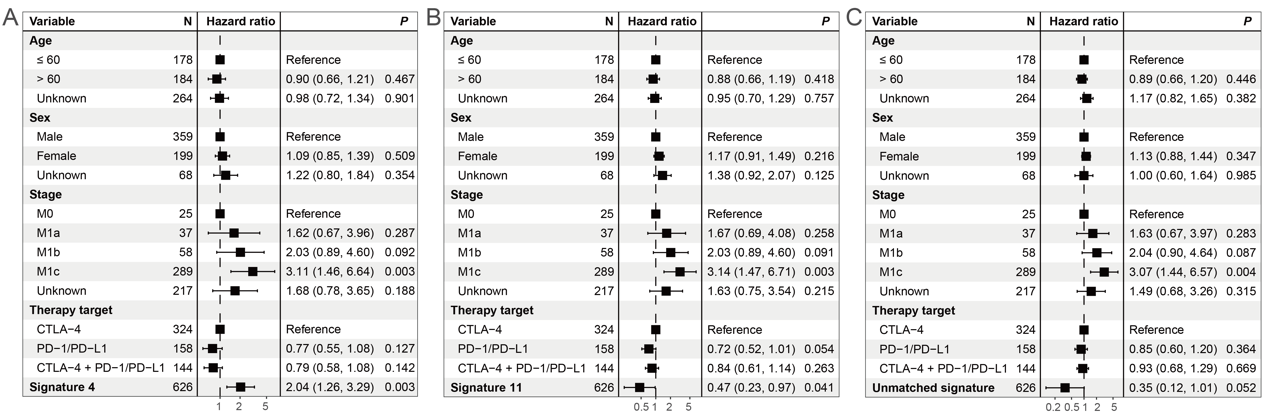


Figure S20. Multivariate Cox regression models depiction of the connections of (A) signature 4, (B) signature 11, and (C) unmatched signature with ICI survival outcome in the setting of treating them as continuous variables.


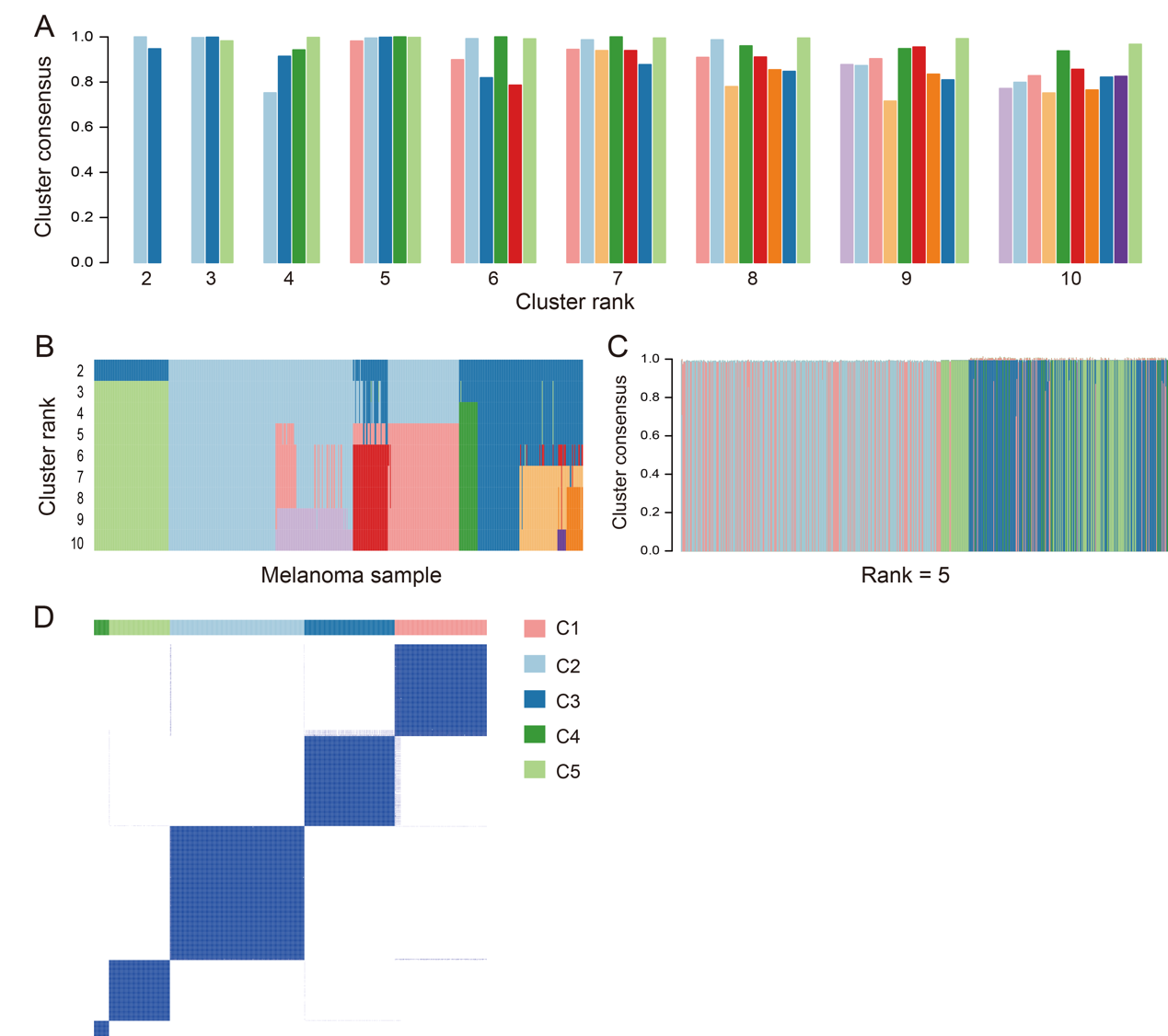


Figure S21. Detailed information of the consensus clustering analysis of mutational signatures activities. (A) Cluster consensus exhibition when the cluster rank was selected as 2 to 10, respectively. (B) The clustering tracking plot illustration under the increase of the cluster number. (C) The clustering consensus with the cluster number was selected as 5. (D) The consensus matrix when the cluster number was selected as 5.


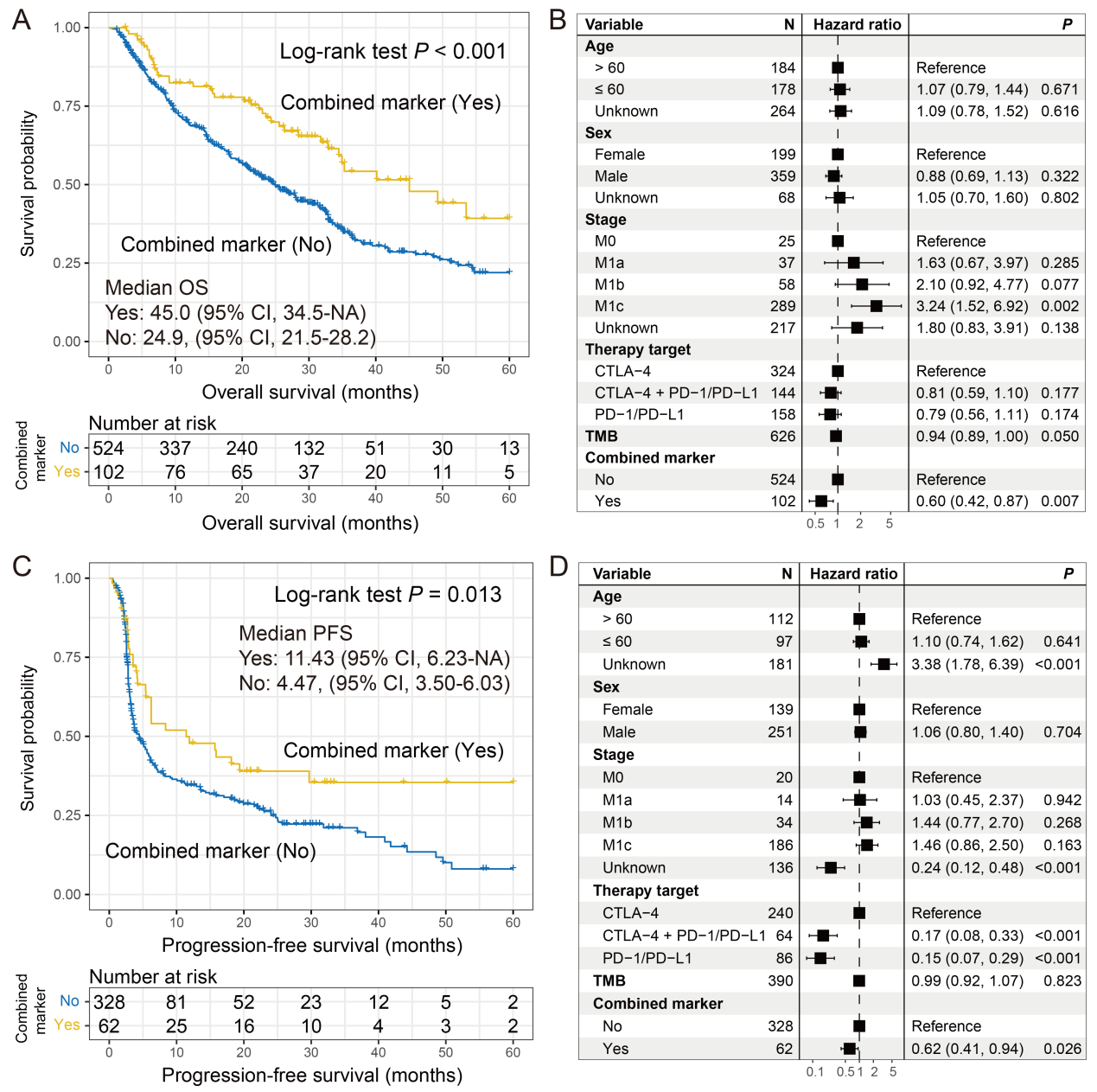


Figure S22. Kaplan-Meier survival analysis and multivariate Cox regression models were conducted to elucidate the associations of the combined marker with (A, B) ICI OS outcome and (C, D) PFS outcome.


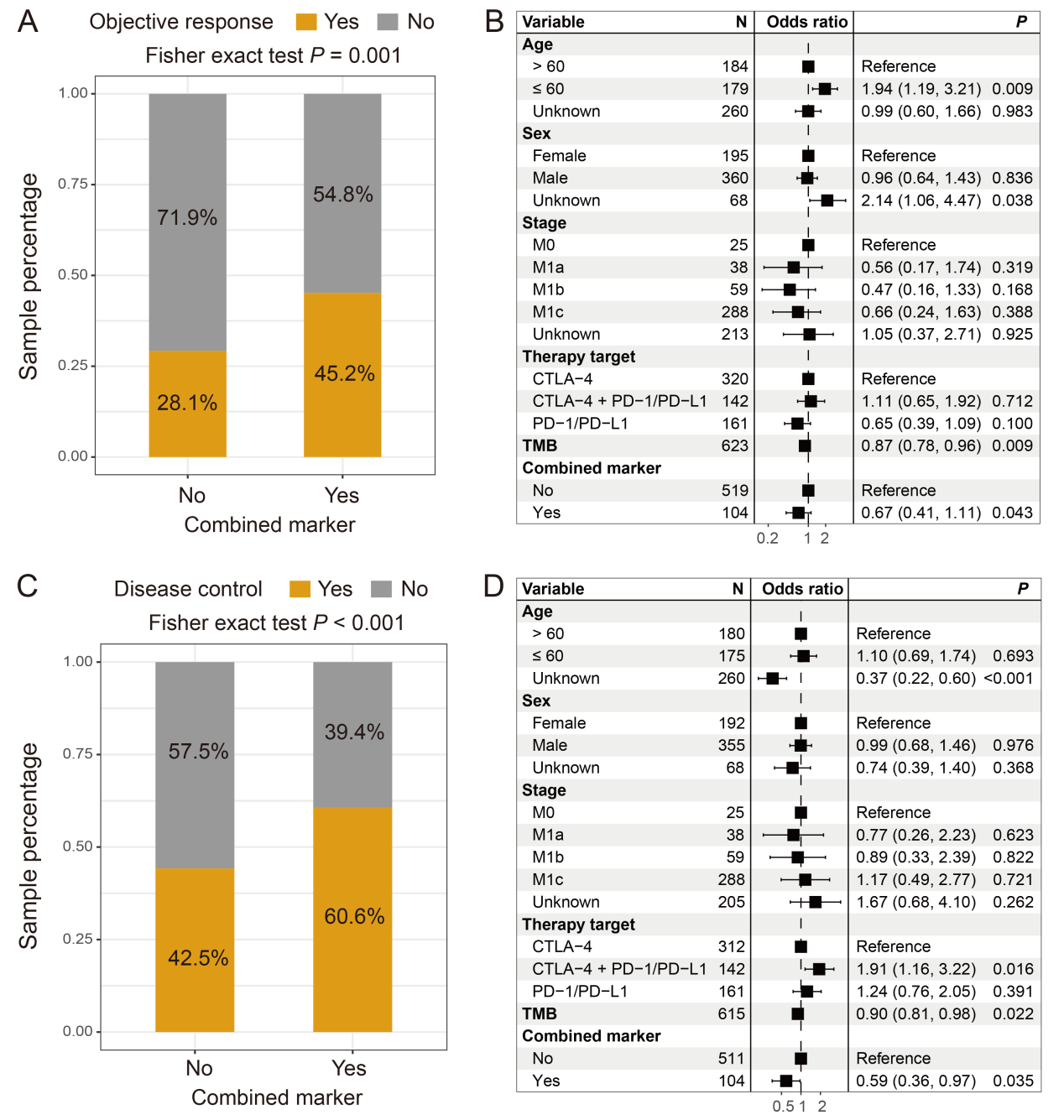


Figure S23. Fisher exact test and multivariate Logistic regression models were conducted to elucidate the associations of the combined marker with (A, B) ORR and (C, D) DCR.
